# Geographical variations in patient-reported outcomes after total hip arthroplasty between 2008 - 2012

**DOI:** 10.1186/s12913-019-4171-5

**Published:** 2019-05-30

**Authors:** Linnea Oldsberg, Göran Garellick, Ingrid Osika Friberg, Anke Samulowitz, Ola Rolfson, Szilárd Nemes

**Affiliations:** 1 Swedish Hip Arthroplasty Register, Gothenburg, Sweden; 2 Department of Public Health and Community Medicine, The Sahlgrenska Academy, University of Gothenburg, Gothenburg, Sweden; 3 Department of Orthopaedics, Institute of Clinical Sciences, The Sahlgrenska Academy, University of Gothenburg, Gothenburg, Sweden; 4 Centre for Equity in Health Care, Region Västra Götaland, Sweden

**Keywords:** Patient-reported outcomes, Total hip arthroplasty, Geographical variations, Equity in health care

## Abstract

**Background:**

Health care on equal terms is a cornerstone of the Swedish health care system. Total hip arthroplasty (THA) is considered a success story in Sweden with low frequency of reoperations and restored health-related quality of life (HRQoL). Administratively, health care in Sweden is locally self-governed by 21 counties. In this longitudinal nation-wide observational study we assessed the possible geographical variations in 1-year follow-up patient-reported outcomes (PROs): EQ-5D index, EQ VAS, Pain VAS and Satisfaction VAS.

**Methods:**

Study population consisted of 36,235 Swedish THA patients, operated during 2008 to 2012 due to hip osteoarthritis. Individual data came from Swedish Hip Arthroplasty Register, Statistics Sweden and National Board of Health and Welfare. We used descriptive statistics together with multivariable regression analysis to analyse the data.

**Results:**

We observed county level differences in both preoperative and postoperative PROs. The results showed that the differences observed in preoperative PROs could not fully explain the differences observed in postoperative PROs, even after adjustment for patient demographics (age, sex, BMI, Elixhauser comorbidity index, marital status, educational level and disposable income). This indicates that other factors might influence the outcome after THA.

**Conclusion:**

Likely, structural and process differences such as indication for surgery have an influence on PROs after surgery. Standardization of care at hospital levels may decrease geographical variations in postoperative HRQoL. Remaining differences will then possibly be associated to patient demographics.

**Electronic supplementary material:**

The online version of this article (10.1186/s12913-019-4171-5) contains supplementary material, which is available to authorized users.

## Background

Health care on equal terms is a cornerstone of the Swedish health care system. The Swedish Association of Local Authorities and Regions has created a national platform to improve equity in health and health care. Equity in health care implies that all citizens should have the same opportunity and access to health care despite e.g. age, sex, socioeconomic status, sexual orientation, ethnicity or place of residence. In Sweden health care is locally self-governed by county councils and regions responsible for providing good health care on equal terms. However, with a decentralised health care, regional health care differences in quality and efficiency can exist [1]. Geographical variations are one of many inequities that health care users may experience. Other examples are age and gender inequalities that by itself, or in combination with geographical variations, can influence the patient’s care.

Osteoarthritis (OA) of the hip is a common joint disorder, causing pain and functional disability. The disease affects all parts of the joint making it difficult to handle daily living, thus negatively affecting health-related quality of life (HRQoL). In Global Burden of Disease 2010, hip and knee OA was categorized as the 11th highest contributor of global disability, posing a significant public health problem [2]. Every fourth person over 45 in Sweden has OA and that will most likely increase, due to an ageing population and a higher number of people with obesity [3]. This will challenge health care and increase societal costs; sick leave due to OA alone costs the social insurance system 1.4 billion SEK annually [1].

A predictable higher incidence of OA will, most likely, increase the demand for total hip arthroplasty (THA) [4]. A growth in demand might, in turn, lead to a greater variation of THA care between counties since the growth will not be linear between counties and the self-govern policies may result in differences in the provision of care. Geographical variations in rate of THA have been explored in several countries. In Finland, the highest-scoring region had almost twice as many THAs compared to the lowest-scoring region [5]. Surgeon decision-making related factors influenced the rate of THA but socioeconomic status was not an explanatory factor. Contrary to this, a study by Judge and collaborators from 2009 observed that in England the incidence of THA in different regions had been influenced by socio-demographic factors [6]. The authors concluded that the rate of THA varied across the country, even after adjustment for distance, socio-demographic and hospital factors. Similar results were observed in Australia by Dixon and collaborators in 2010, with 13% lower rates of THA in males and 18% lower rates in females in the most, compared to the least, socioeconomically weak area [7].

Sweden has a well-known history of high quality health care. In the field of THA, Sweden is a leading country with low figures in reoperations, adverse events and mortality. THA is a common procedure in Sweden, with around 17,000 surgeries per year. OA is the primary cause of THA, 85% of male THA patients have OA; the corresponding figure for women is 80%. Six out of ten THA patients are women and the average age when undergoing THA is higher for women (70 years) than for men (67,3 years). The Swedish Hip Arthroplasty Register (SHAR) has registered THAs in Sweden since 1979, currently with 100% hospital coverage and 98% completeness for primary THAs. SHAR collects patient-reported outcome measures (PROMs), with the purpose of understanding the outcome from a patient’s perspective [8, 9]. Most patients undergo THA due to pain, reduced mobility and low HRQoL. Consequently, patient-reported measures in SHAR include EQ-5D, EQ VAS, hip pain and patient satisfaction with the outcome of surgery. Several articles have stated differences in patient-reported outcomes (PROs) after THA in equity factors like sex, age and socioeconomic status. Female sex, higher age, low educational level, more comorbidities, higher BMI and lower income have all been associated with lower PROs [10–12]. Surgical approach also seems to have an effect on PROs after THA [13, 14]. Thus, some inequities in PROs after THA have already been confirmed but the geographical differences in PROs are at this point unknown.

The aim of this nationwide observational registry study was 1) to explore geographical variations in patient-reported outcomes in total hip arthroplasty and 2) to determine to what extent potential geographical variations are explained by patient-related and socioeconomic variables.

## Methods

### Study population

**Fig. 1:**
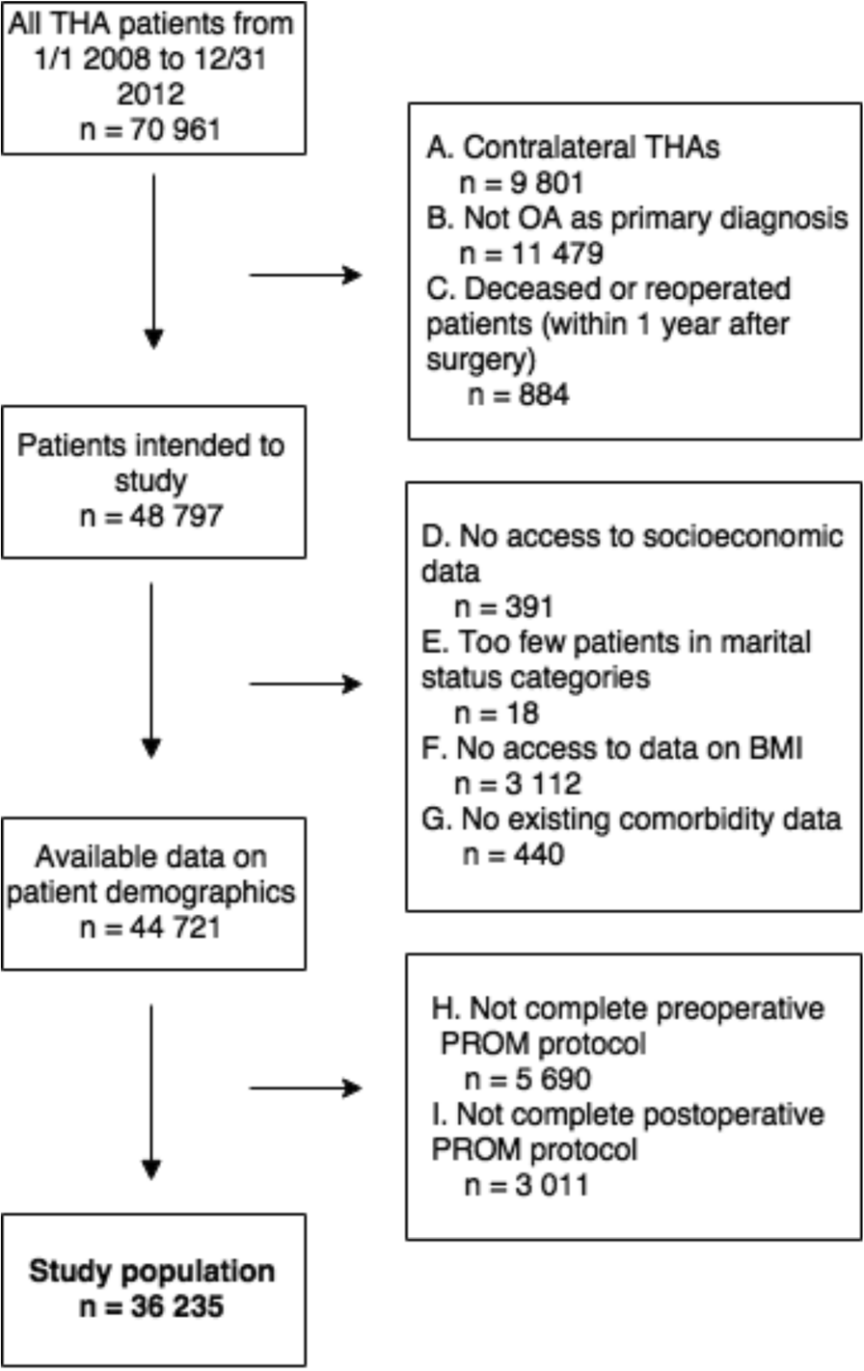
Flowchart over study population

This study consisted of elective primary THA patients operated in Sweden between 1st of January 2008 and 31st of December 2012. Thus, the follow-up ended 31st of December 2013. Patients included in the analysis had primary OA and completed self-reported PROM protocols before and 1 year after THA. This is a standardised protocol used by SHAR primarily quality control of the Swedish heath care system and secondly for research purposes [15]. If patients had bilateral THAs during the study period, only the first THA was included in the analysis. Access to data on socioeconomic variables, marital status, comorbidities and BMI was necessary for inclusion. The study population included 36,235 patients, 56.3% women and 43.7% men, see flowchart (Fig. 1) for more details.

### Data sources

Data for patients identified in the SHAR data base were linked to Statistics Sweden and The National Board of Health and Welfare’s patient register using the unique 10-digit Personal Identification Number [14]. SHAR provided data on BMI and pre- and 1 year postoperative PRO values, with a completeness of 75%. Preoperative PROMs consisted of EQ-5D-3 L, EQ VAS and pain VAS. Postoperative PROMs contained the above, as well as patient satisfaction VAS. In this study EQ-5D-3 L will be referred to as EQ-5D. EQ-5D is a common generic instrument for HRQoL and can be supplemented with EQ VAS, which registers the respondent’s self-rated health on a vertical, visual analogue scale [16]. For calculating the EQ-5D the British value set was used, where the index value rank from − 0.594 to 1 [17]. Patient satisfaction, pain and EQ VAS are measured on VAS scales from 0 to 100. Higher values of EQ-5D and EQ VAS indicate better results. In contrary, higher values of pain and satisfaction VAS indicate worse results. Statistics Sweden provided individual data on educational level, disposable income, marital status, age and sex. The National Board of Health and Welfare’s patient register was used for data on comorbidities.

### Variables

Patient-related variables were sex, age, marital status, comorbidities and BMI. Socioeconomic variables consisted of disposable income and educational level. Educational level was divided into three categories; primary education (≤10 years), upper secondary education (10–12 years) and higher education (≥3 years in university). Marital status was categorized as unmarried, married, divorced and widow/widower. Elixhauser comorbidities index is a comprehensive set of 31 comorbidity measures [18]. Elixhauser comorbidities index, disposable income, BMI and age were continuous variables in the analysis, to maintain predictive power [19]. In Sweden county councils and regions have health care responsibilities; for simplicity all 21 will be called counties in this study.

### Statistical analysis

#### Descriptive statistics

We used descriptive statistics to analyse if geographical variations exist in PROs in THA patients, on county level. Continuous data was summarized as means ±1 standard deviation, categorical data as counts and percentages. Graphical presentation of the PRO values is based on the mean values at county levels. First, we present observed preoperative PROs data, second, we present the observed postoperative data. Difference between pre- and postoperative PRO values is presented as absolute differences (delta) and Improvement Ratio (IR) [20]. The delta values give a direct interpretable measure of the attained improvement. However, the improvement is largely dependent on the preoperative value (e.g. a 10-unit pain relief could mean from 10 to 0 or from 60 to 50). The IR measures the percentage of the achieved improvement of the total possible improvement, thus factoring in the preoperative value (e.g a 10-unit pain relief could mean from 10 to 0 result in an IR value of 100, while 60 to 50 giving an IR value of 16%). Summary statistics are presented for the final study population.

#### Regression analyses

Several factors influence the observed postoperative PROs, for example surgical techniques and implant choice which are modifiable factors. Other factors such as educational level, disposable income, marital status, BMI, age, sex, comorbidities and preoperative PROs are unmodifiable by the surgeon or hospitals. To consider these unmodifiable factors a series of multivariable regression analyses were conducted. We regressed the postoperative PRO values on the above listed un-modifiable factors. To counteract the possible bias induced by missing data points we conducted Multiple Imputations by Fully Conditional Specifications (FCS) [21] using the ‘mice’ R package [22]. Regression coefficients and linear predictors per patient were combined using Rubin’s rules [23]. Thereafter we calculated the difference between the observed and expected postoperative PROs (i.e. regression residuals) that represent the variability in postoperative PROs not explained by the considered factors.

Predictive power was summarized as coefficient of determination (R^2^) which measures the amount of observed variability of the outcome explained by the exposure and co-variates. The partial-R^2^ gives the individual contribution of each variable to the final predictive power.

Preoperative, postoperative and the differences between the observed and expected preoperative PROs was divided into three groups, where counties with national average PRO values ± one standard deviation gave the middle bracket, represented in blue colour. Lower bracket, in red colour, indicated divergence with at least one standard deviation from the national mean into negative direction. Higher bracket, in green colour, indicated PRO results that are better than at least one standard deviation from the national mean. Hence, pain and satisfaction VAS scales are inverted for consistency.

Coefficients in a linear regression model is measured in the same units as the outcome. Thus, the regression coefficients should be interpreted as the adjusted deviation from the reference value. Västmanland, the county closest to national mean values was used as a reference value. For example, the reference value for patient satisfaction is 7.93. Västerbotten county has a regression coefficient of −.76, meaning that the patient satisfaction in this county is 7.17. However, as the confidence interval supports an increase of patient satisfaction with 2.41 units and a decrease with 0.89 we have no support that patient satisfaction in Västerbotten county deviates from the reference.

#### Ethics

The data collection and preparation of this article has been carried out in accordance with the ethical principles for medical research involving human subjects of the World Medical Association’s Declaration of Helsinki [24]. The study was approved by the regional ethical review board in Gothenburg, Medical Department, April 7, 2014 (dnr 271–14).

## Results

**Fig. 2:**
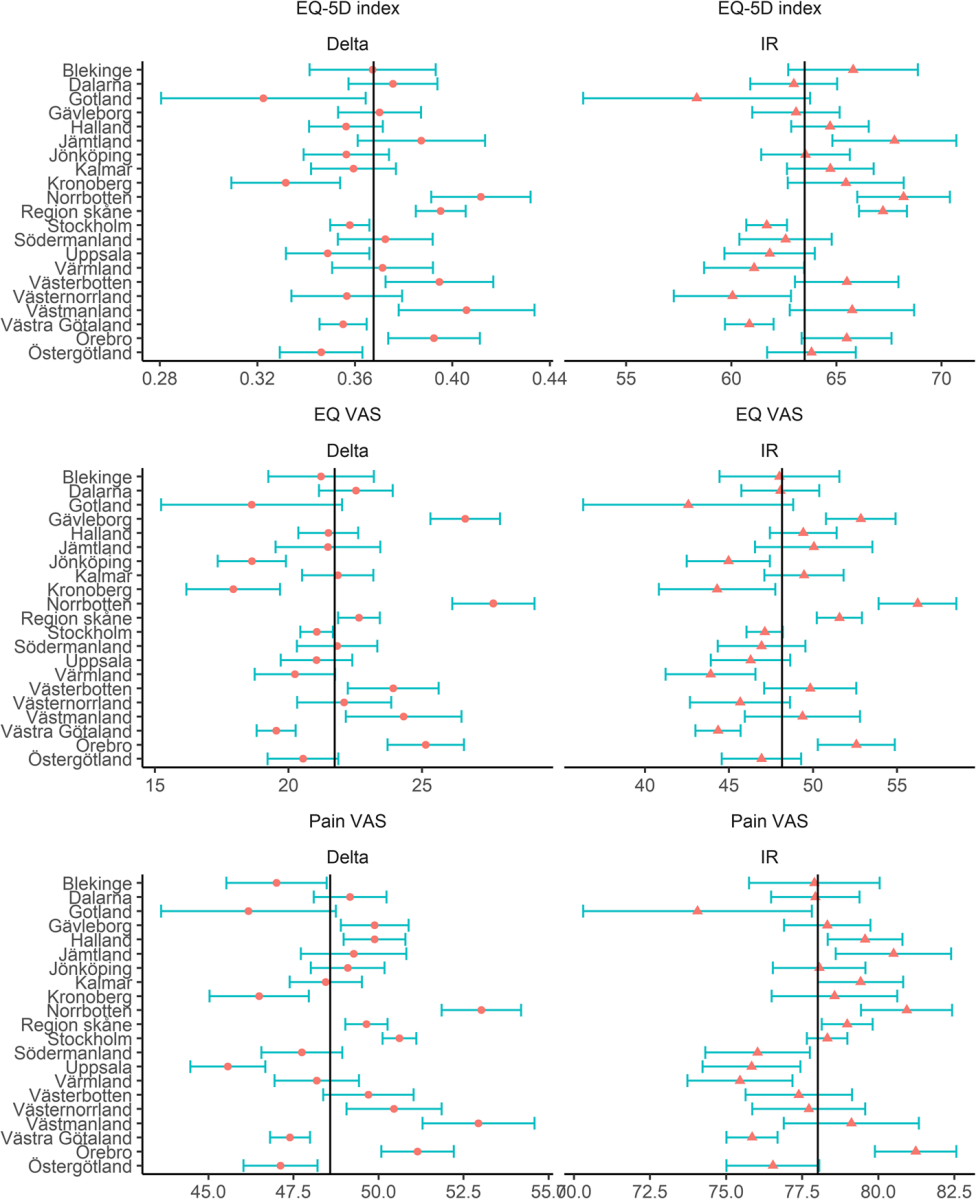
Realised differences between pre- and post-operative PRO values (EQ-5D index, EQ VAS and Pain VAS) for the 21 Swedish counties (Delta) and the Improvement Ratio index (IR) that measures the achieved improvement as a percentage of the total possible improvement

**Fig. 3:**
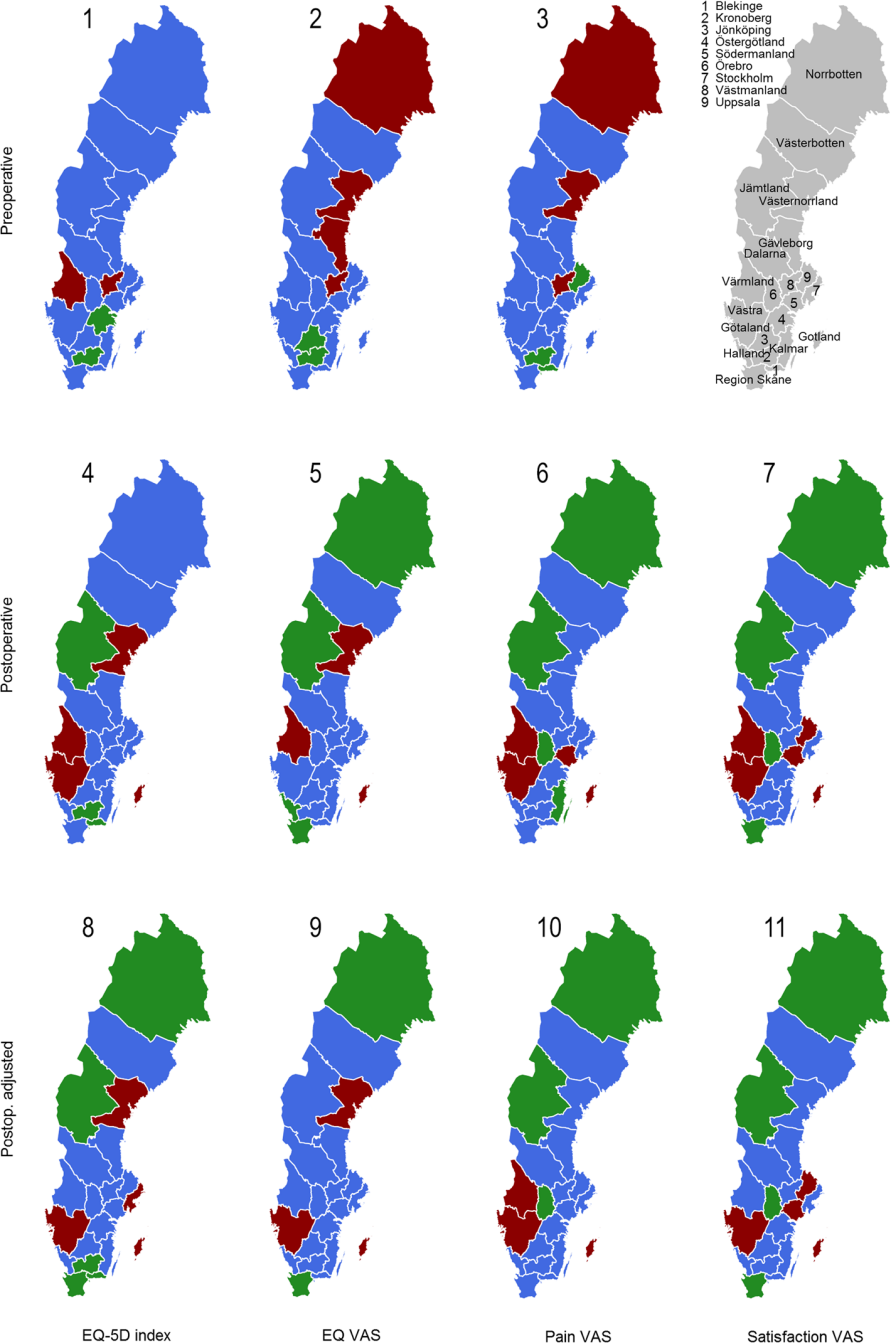
Geographical variations on county level in PROs before and after THA. Legend: Map 1–7 show observed pre- and postoperative PROS. Map 8–11 show adjusted postoperative results (controlled for age, sex, marital status, BMI, comorbidities, disposable income, educational level and preoperative PRO values). Blue colour represent counties with national average PRO values ± one standard deviation. Red colour indicates divergence with at least one standard deviation from the national mean into negative direction. Green colour indicates PRO results that are better than at least one standard deviation from the national mean

Table 1 gives background characteristics on study population, divided on county level. The biggest improvement was seen in pain relief, thereafter in EQ-5D index and EQ VAS (Fig. 2). Figure 3 shows 11 Swedish maps with geographical variations in PROs, on county level. The R computing environment was used to generate the maps presented. The maps were coded by the authors and are based on routines and R packages in the public domain. Three maps (1, 2 and 3) show variations in observed preoperative PROS, four maps (4, 5, 6 and 7) give variations in observed 1 year postoperative PROs, and four maps (8, 9, 10 and 11) present variations in adjusted postoperative PRO values. As seen, postoperative PRO values (map 4, 5, 6, and 7) did not always conform to preoperative PRO values (map 1, 2 and 3) in the same county. Map 1 shows the preoperative EQ-5D index and indicates that almost all counties had average preoperative EQ-5D values ± one standard deviation from the national mean, illustrated in blue. Two counties had poorer preoperative EQ-5D values than one standard deviation from the mean, illustrated in red. Two counties had more favourable preoperative EQ-5D values than one standard deviation from the mean, marked with green colour. Map 4 illustrates the postoperative EQ-5D index; here seven counties had better or worse results than one standard deviation from the mean.

**Table 1:** Background characteristics of each county, average of 2008–2012

County	Blekinge	Dalarna	Gotland	Gävleborg	Halland	Jämtland	Jönköping
Total n	665	1452	249	1614	2072	636	1404
Sex, n female (%)	353 (53.1)	785 (54.1)	130 (52.2)	854 (52.9)	1150 (55.5)	372 (58.5)	788 (56.1)
mean Age (sd)	68.6 (9.7)	68.6 (9.5)	67.8 (9.2)	67.5 (9.8)	68.0 (9.6)	69.0 (9.4)	68.7 (10.0)
mean BMI (sd)	27.3 (4.6)	27.8 (4.8)	28.5 (12.7)	27.7 (4.7)	27.1 (6.0)	27.3 (4.3)	27.7 (8.1)
mean Elixhauser (sd)	1.06 (1.03)	0.92 (1.02)	0.38 (0.70)	0.65 (0.97)	0.66 (0.97)	0.52 (0.93)	0.99 (1.06)
Marital status (%)							
married	383 (57.6)	798 (55.0)	140 (56.2)	894 (55.4)	1293 (62.4)	339 (53.3)	904 (64.4)
unmarried	77 (11.6)	180 (12.4)	30 (12.0)	210 (13.0)	167 (8.1)	90 (14.2)	125 (8.9)
divorced	99 (14.9)	262 (18.0)	39 (15.7)	288 (17.8)	324 (15.6)	102 (16.0)	170 (12.1)
widow/widower	106 (15.9)	212 (14.6)	40 (16.1)	222 (13.8)	288 (13.9)	105 (16.5)	205 (14.6)
Education attainment (%)							
primary	231 (34.7)	539 (37.1)	94 (37.8)	609 (37.7)	734 (35.4)	212 (33.3)	632 (45.0)
upper secondary	281 (42.3)	660 (45.5)	100 (40.2)	671 (41.6)	834 (40.3)	285 (44.8)	528 (37.6)
higher	153 (23.0)	253 (17.4)	55 (22.1)	334 (20.7)	504 (24.3)	139 (21.9)	244 (17.4)
median disposable income/year SEK (mean)	153,200 (178178)	148,600 (176903)	146,300 (174769)	156,800 (188098)	166,450 (213306)	147,150 (181821)	152,950 (193048)
Preoperative PROs							
mean EQ-5D (sd)	0.44 (0.32)	0.40 (0.31)	0.45 (0.30)	0.41 (0.32)	0.45 (0.31)	0.43 (0.30)	0.44 (0.30)
mean EQ VAS (sd)	55.8 (21.5)	53.1 (22.3)	56.3 (21.6)	49.6 (21.9)	56.5 (22.7)	57.1 (21.8)	58.6 (20.4)
mean pain VAS (sd)	59.1 (15.8)	62.2 (16.1)	60.6 (16.5)	63.0 (14.5)	61.9 (16.5)	61.0 (16.0)	61.8 (15.3)
Postoperative PROs							
mean EQ-5D (sd)	0.81 (0.23)	0.78 (0.23)	0.77 (0.24)	0.78 (0.25)	0.81 (0.23)	0.82 (0.21)	0.80 (0.22)
mean EQ VAS (sd)	77.0 (20.1)	75.7 (20.2)	74.9 (20.1)	76.3 (21.3)	78.0 (19.5)	78.6 (18.7)	77.2 (18.7)
mean pain VAS (sd)	13.1 (16.7)	13.7 (17.7)	15.7 (18.3)	13.7 (18.6)	12.7 (17.8)	11.9 (15.1)	13.6 (17.9)
mean satisfaction VAS (sd)	14.6 (19.1)	15.9 (20.3)	19.1 (22.3)	15.4 (20.8)	14.7 (20.9)	13.1 (17.2)	15.3 (18.9)
County	Kalmar	Kronoberg	Norrbotten	Skåne	Stockholm	Södermanland	Uppsala
Total n	1356	741	1102	4180	7075	1183	1398
Sex, n female (%)	745 (54.9)	379 (51.1)	624 (56.6)	2382 (57.0)	4235 (59.9)	678 (57.3)	764 (54.6)
mean Age (sd)	68.0 (9.7)	68.4 (10.3)	68.1 (9.6)	68.5 (10.3)	67.4 (10.2)	68.2 (9.8)	67.5 (9.9)
mean BMI (sd)	27.7 (4.6)	27.3 (4.1)	27.4 (4.3)	27.4 (5.7)	26.8 (5.0)	27.4 (4.2)	27.0 (4.3)
mean Elixhauser (sd)	0.60 (0.95)	0.74 (0.99)	0.93 (1.07)	1.11 (1.17)	0.54 (0.93)	0.81 (0.99)	0.87 (1.09)
Marital status (%)							
married	825 (60.8)	466 (62.9)	668 (60.6)	2462 (58.9)	3793 (53.6)	688 (58.2)	829 (59.3)
unmarried	139 (10.3)	75 (10.1)	111 (10.1)	365 (8.7)	882 (12.5)	91 (7.7)	147 (10.5)
divorced	176 (13.0)	85 (11.5)	160 (14.5)	709 (17.0)	1443 (20.4)	205 (17.3)	236 (16.9)
widow/widower	216 (15.9)	115 (15.5)	163 (14.8)	644 (15.4)	957 (13.5)	199 (16.8)	186 (13.3)
Education attainment (%)							
primary	563 (41.5)	272 (36.7)	359 (32.6)	1445 (34.6)	1610 (22.8)	437 (36.9)	434 (31.0)
upper secondary	512 (37.8)	317 (42.8)	502 (45.6)	1647 (39.4)	2933 (41.5)	488 (41.3)	520 (37.2)
higher	281 (20.7)	152 (20.5)	241 (21.9)	1088 (26.0)	2532 (35.8)	258 (21.8)	444 (31.8)
median disposable income/year SEK (mean)	154,150 (199187)	154,000 (206228)	153,500 (183822)	162,200 (213102)	194,400 (268747)	156,700 (187498)	176,800 (257385)
Preoperative PROs							
mean EQ-5D (sd)	0.44 (0.31)	0.49 (0.30)	0.40 (0.31)	0.41 (0.31)	0.42 (0.32)	0.40 (0.32)	0.44 (0.31)
mean EQ VAS (sd)	55.9 (21.8)	59.5 (20.3)	50.8 (22.7)	56.1 (22.7)	55.3 (22.1)	53.5 (21.5)	54.5 (21.6)
mean pain VAS (sd)	60.6 (15.7)	58.6 (15.7)	64.9 (14.6)	62.3 (15.7)	63.7 (16.3)	61.7 (15.5)	59.4 (15.5)
Postoperative PROs							
mean EQ-5D (sd)	0.80 (0.22)	0.82 (0.19)	0.81 (0.22)	0.81 (0.22)	0.78 (0.24)	0.78 (0.23)	0.78 (0.24)
mean EQ VAS (sd)	77.7 (19.4)	77.5 (18.5)	78.5 (18.9)	78.7 (18.9)	76.4 (20.0)	75.3 (20.5)	75.6 (20.1)
mean pain VAS (sd)	12.5 (16.1)	12.6 (16.9)	12.4 (16.5)	13.1 (17.4)	13.8 (18.2)	14.8 (18.7)	14.4 (18.4)
mean satisfaction VAS (sd)	13.8 (18.2)	14.0 (19.3)	13.3 (17.1)	13.3 (18.9)	16.4 (21.9)	18.3 (22.3)	17.4 (22.3)
County	Värmland	Västerbotten	Västernorrland	Västmanland	Västra Götaland	Örebro	Östergötland
Total n	1109	979	900	653	4742	1327	1398
Sex, n female (%)	624 (56.3)	552 (56.4)	531 (59.0)	358 (54.8)	2623 (55.3)	726 (54.7)	751 (53.7)
mean Age (sd)	69.2 (9.7)	67.4 (9.9)	68.4 (8.8)	68.2 (9.5)	68.1 (10.3)	67.6 (9.6)	68.3 (10.2)
mean BMI (sd)	27.7 (4.5)	27.4 (7.3)	27.9 (7.4)	28.2 (4.6)	27.3 (4.4)	27.6 (4.6)	27.4 (4.4)
mean Elixhauser (sd)	0.88 (1.05)	1.05 (1.10)	0.60 (0.93)	0.81 (1.03)	0.66 (0.97)	0.76 (0.97)	0.83 (0.99)
Marital status (%)							
married	608 (54.8)	579 (59.1)	506 (56.2)	384 (58.8)	2802 (59.1)	792 (59.7)	830 (59.4)
unmarried	141 (12.7)	121 (12.4)	104 (11.6)	71 (10.9)	470 (9.9)	131 (9.9)	155 (11.1)
divorced	165 (14.9)	136 (13.9)	153 (17.0)	97 (14.9)	757 (16.0)	207 (15.6)	190 (13.6)
widow/widower	195 (17.6)	143 (14.6)	137 (15.2)	101 (15.5)	713 (15.0)	197 (14.8)	223 (16.0)
Education attainment (%)							
primary	459 (41.4)	326 (33.3)	321 (35.7)	237 (36.3)	1820 (38.4)	473 (35.6)	528 (37.8)
upper secondary	448 (40.4)	414 (42.3)	400 (44.4)	296 (45.3)	1847 (38.9)	572 (43.1)	578 (41.3)
higher	202 (18.2)	239 (24.4)	179 (19.9)	120 (18.4)	1075 (22.7)	282 (21.3)	292 (20.9)
median disposable income/year SEK (mean)	146,000 (172373)	157,900 (189558)	148,850 (181142)	156,000 (187402)	157,200 (213276)	158,900 (192642)	159,250 (200990)
Preoperative PROs							
mean EQ-5D (sd)	0.39 (0.31)	0.40 (0.31)	0.41 (0.31)	0.38 (0.33)	0.42 (0.31)	0.40 (0.32)	0.46 (0.30)
mean EQ VAS (sd)	53.9 (21.6)	52.0 (23.1)	51.6 (22.0)	50.8 (23.4)	55.9 (21.3)	52.2 (22.5)	56.2 (22.3)
mean pain VAS (sd)	63.0 (15.2)	63.4 (15.7)	64.5 (14.8)	66.0 (14.8)	61.3 (16.0)	62.5 (15.9)	60.8 (15.8)
Postoperative PROs							
mean EQ-5D (sd)	0.76 (0.24)	0.79 (0.23)	0.76 (0.26)	0.79 (0.23)	0.77 (0.24)	0.79 (0.24)	0.80 (0.22)
mean EQ VAS (sd)	74.1 (20.5)	75.9 (20.2)	73.7 (21.5)	75.1 (21.1)	75.5 (20.1)	77.3 (19.6)	76.8 (19.4)
mean pain VAS (sd)	15.5 (18.6)	14.3 (17.7)	14.4 (18.4)	13.8 (19.1)	14.8 (18.2)	11.7 (15.6)	14.3 (17.9)
mean satisfaction VAS (sd)	17.8 (21.7)	15.0 (18.5)	16.8 (21.5)	15.6 (20.7)	17.6 (21.6)	13.2 (18.3)	16.1 (20.1)

Legend: Higher values of EQ-5D and EQ VAS indicate better results. In contrary, higher values of pain and satisfaction VAS indicate worse results

Map 3 shows preoperative pain on a VAS scale. Norrbotten, Västernorrland and Västmanland had poorer preoperative pain values than the national mean, marked in red. Patients from Uppsala, Blekinge and Kronoberg had favourable preoperative pain values, illustrated in green colour. Conversely, Norrbotten had a more favourable postoperative pain value than the mean (map 6), together with Jämtland, Örebro and Kalmar. Four counties had poorer postoperative pain values than the mean.

Maps 8, 9, 10 and 11 present postoperative PRO values with adjustment for age, sex, marital status, comorbidities, BMI, education, disposable income and preoperative PRO values. One county had better average values on all adjusted postoperative PROs (green colour). Two counties achieved better results in three out of four adjusted PRO values. Two counties deviated negatively on all postoperative PROs (red colour) after adjustment for socioeconomic and patient-related (including preoperative PROs) variables.

This observed variability cannot be contributed to random variation (Fig. 3).

Multivariable analysis (Table 2) demonstrated that geographical variations exist on county level, even after adjustment for socioeconomic and patient-related variables. Gotland had significantly poorer EQ-5D (β = − 0.04, CI -0.07; − 0.01) compared to Västmanland, while differences in EQ VAS were not significant. Moreover, patients in Gotland had more pain (β = 2.78, CI -1.11; 1.57) and were less satisfied (β = 4.84, CI 2.29; 7.4) with the surgery, compared to the reference values. Norrbotten had significant more favourable results on EQ VAS (β =2.67, CI 1.07; 4.27) and satisfaction (β = − 1.98, CI -3.71; − 0.24) compare to Västmanland, same was true for Skåne for EQ VAS (β = 3,3, CI 1.98; 4.62) and satisfaction (β = − 1.99, CI -3.41; − 0.58). Patients in Stockholm had lower EQ-5D (β = − 0.03, C -0.04; − 0.01I) and were less satisfied (β = 1.87, CI 0.48; 3.26) 1 year after the surgery. Södermanland had no significant postoperative PRO values except a poorer patient satisfaction value (β = 2.5, CI 0.87; 4.14). Uppsala, Värmland and Västra Götaland had significantly less favourable results on satisfaction VAS. Furthermore, Västra Götaland had poorer EQ-5D (β = − 0.03, CI -0.05; − 0.01) than the reference county.

**Table 2:** The effect of county on postoperative PROs

	EQ-5D		EQ VAS		Pain VAS		Satisfaction VAS	
County	Coefficient	95% CI	Coefficient	95% CI	Coefficient	95% CI	Coefficient	95% CI
*Reference*	0.85	0.79; 0.9	81.98	77.17; 86.8	8.54	4.52; 12.56	7.93	2.17; 11.9
Blekinge	0.01	−0.02; 0.03	1.32	− 0.48; 3.11	0.23	4.52; 12.56	−0.41	−2.37; 1.55
Dalarna	−0.01	−0.03; 0.01	0.64	−0.95; 2.23	0.23	−1.42; 1.89	0.63	− 1.04; 2.29
Gotland	−0.04	−0.07; − 0.01	−2.38	−4.77; 0.01	2.78	− 1.11; 1.57	4.84	2.29; 7.4
Gävleborg	−0.01	−0.03; 0.01	0.69	−0.82; 2.2	0.17	0.58; 4.98	0.26	−1.37; 1.89
Halland	0.00	−0.02; 0.02	1.39	−0.01; 2.79	−0.27	−1.21; 1.55	− 0.09	− 1.62; 1.44
Jämtland	0.01	−0.02; 0.03	1.39	−0.46; 3.24	− 0.63	−1.6; 1.06	−1.57	−3.54; 0.41
Jönköping	0.00	−0.02; 0.02	1.11	−0.43; 2.64	0.09	−2.4; 1.13	0.1	−1.54; 1.74
Kalmar	0.00	−0.02; 0.02	1.13	−0.45; 2.7	−0.52	−1.31; 1.49	− 0.96	−2.6; 0.67
Kronoberg	−0.01	− 0.03; 0.02	−0.23	−1.96; 1.5	0.41	−1.92; 0.89	−0.17	−2.01; 1.67
Norrbotten	0.01	−0.01; 0.03	2.67	1.07; 4.27	−1.23	−1.2; 2.01	− 1.98	−3.71; −0.24
Skåne	0.01	0.00; 0.03	3.3	1.98; 4.62	−0.41	−2.62; 0.16	−1.99	−3.41; − 0.58
Stockholm	− 0.03	− 0.04; − 0.01	−0.61	−1.91; 0.69	1.1	−1.68; 0.86	1.87	0.48; 3.26
Södermanland	−0.02	−0.04; 0.00	0.05	−1.51; 1.62	1.11	−0.04; 2.24	2.5	0.87; 4.14
Uppsala	−0.02	−0.04; 0.00	− 0.44	−1.98; 1.1	1.62	− 0.33; 2.55	2.76	1.16; 4.36
Värmland	−0.02	−0.04; 0.00	− 0.73	−2.34; 0.88	1.78	0.24; 3	2.47	0.82; 4.13
Västerbotten	0.01	−0.01; 0.02	1.07	−0.51; 2.64	0.49	0.3; 3.25	−0.76	−2.41; 0.89
Västernorrland	−0.02	−0.04; 0.00	−1.26	−2.91; 0.39	0.74	−0.94; 1.91	1.29	−0.56; 3.14
Västra Götaland	−0.03	−0.05; − 0.01	−0.82	−2.13; 0.48	1.45	−0.91; 2.39	2.52	1.12; 3.91
Örebro	0.00	−0.02; 0.02	1.5	−0.01; 3.01	−1.62	0.23; 2.66	−1.69	−3.54; 0.17
Östergötland	0.00	−0.02; 0.02	0.76	−0.79; 2.31	0.8	−3.05; −0.2	1.05	−0.56; 2.65

Legend: Multivariable analysis shows the effect of county on postoperative PROs, adjusted for sex, age, BMI, Elixhauser comorbidity index, marital status, educational level, disposable income and preoperative PROs. Västmanland (county closest to national mean values) was used as a reference value. Higher values of EQ-5D and EQ VAS indicate better results. In contrary, higher values of pain and satisfaction VAS indicate worse results

**Fig. 4:**
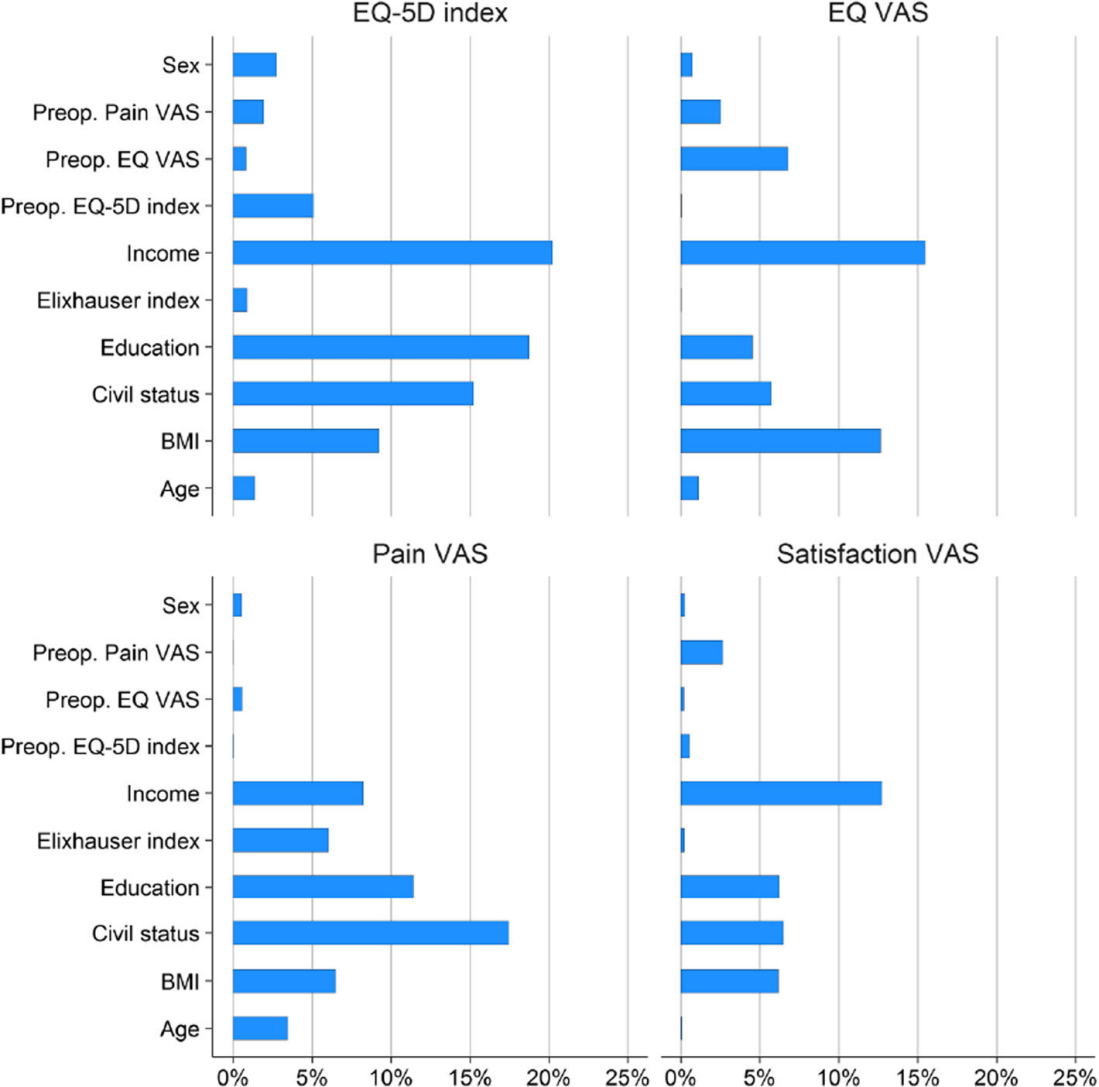
Percentage of explained county-wise variance of the one-year postoperative PROs by the considered 3 covariates

The models explained low amount of variability at patient level (EQ-5D index 9.5%, EQ VAS 10.1%, Pain VAS 3.6% and Satisfaction VAS 2.9%). The predictive power at county level was high with 66.5% of the county-wise variation of the EQ-5D index was explained by the considered co-variates, 46.1% for the EQ VAS, 37.2 for Pain VAS and 35.3 for Satisfaction VAS (Fig. 4).

## Discussion

In this longitudinal register study, we demonstrated that geographical variations in PROs 1 year after THA exist. The results also showed that average PRO values in one county 1 year after THA not always conformed to average PRO values in that same county before surgery. Socioeconomic and patient-related variables, did not fully explain the geographical variations. Nonetheless, the results also showed that socioeconomic and patient-related variables influence PROs 1 year after surgery, which agrees with previous studies [10–12]. Similar results were obtained by Mindemark and collaborators [25] whom examined inter-county variations in laboratory tests in Sweden. The authors found that large variations between all studied counties that cannot traced back to demographic variations but are likely influenced by regional habits and traditions. Caesarean section rate variation between Swedish hospitals are not explained by patient case mix either [26]. On the other hand Broberg and collaborators [27] concluded that both county of residence and socio-economic factors were strongly associated with lower attendance in cervical screening. Regional differences in clinical practice including adherence to treatment guidelines are thought to contribute to regional disparities in rheumatoid arthritis and gout hospitalizations in Sweden [28]. Hospitalizations due to hip osteoarthritis are on rise in Sweden with an attenuated regional disparities for women [29]. Thus, it is likely that health care practices, administrative differences play a role in the observed postoperative PRO variations.

Gotland and Västra Götaland had a negative divergence from national mean on all adjusted postoperative PRO values. Patients operated in Stockholm, Södermanland, Uppsala, Västernorrland and Värmland also deviated negatively on at least one of the adjusted postoperative PRO values. However, 18 out of 21 counties had better results, or results within the national mean range, on one or more adjusted postoperative PRO values, indicating a positive THA health care. Sweden is one of the leading countries in THA hence, the geographical differences in PROs on county level are small but still important in order to improve and support counties that do not achieve as good results as their peers. While we attained statistical significance, we cannot state clinical significance. We believe that almost all patients considered had a clinically significant improvement of their HRQoL. However, as the county level differences exists and show a persistent time trend (Additional file 1) we trust that these recorded differences should be addressed as negative deviation from the country average could indicate a large number of patients with insufficient improvement and dissatisfied with the results of the operation.

It is important to adjust for preoperative PROs when analysing postoperative PROs since it is the factor with the strongest ability to predict the postoperative outcomes [30]. Preoperative PRO values, presented in map 1 to 3, show that variations in patients’ HRQoL and pain existed even before the surgery. Patients in some counties had more pain and lower HRQoL before the surgery, indicating that the patients had lived with OA for a longer time period before seeking, or getting, the care they needed. Other counties showed preoperative values of less pain and higher HRQoL than the national average, which might represent patients who got operated early in their OA process.

Studies from other countries have shown geographical variations in rate of THA [5–7] and in PROs after joint surgery [31]. Since SHAR has access to national coverage data on PROs after THA, we can analyse geographical variations from a patient’s perspective. To our knowledge, this is the first study of its kind, which makes the results important, not least to policy makers. The Swedish Association of Local Authorities and Regions has concluded that there are several explanations behind geographical differences in health care in Sweden today. It could both be demographic and morbidity differences, together with characteristics on health care structure and doctors’ approach and attitude towards patients [32]. The results indicate that a decentralised orthopaedic health care can lead to differences in the care process between counties that might affect patients’ well-being after surgery. Patients in Norrbotten, a county with long distance to the nearest hospital, had lower HRQoL and more pain before surgery. One possible explanation could be altered care-seeking behaviours; we could assume that patients choose to live with pain for a longer time period depending on distance to closest hospital. Lower physician density and diagnostic activity in northern Sweden is thought to be one of the reasons of higher incidences abdominal aortic aneurysm in the norther regions compared mid and south Sweden [33]. Bolin and collaborators [34] concluded that there are significant variation in the prevalence of epilepsy and the provision of health care for patients with epilepsy across the different regions of Sweden, and that geographical distances to advanced healthcare services do not seem to explain these results. While THA and epilepsy is not directly comparable this could be the case for THA patients as well. Another possible reason could be that hospitals in the north of Sweden wait longer with surgery than in the south of Sweden, which should be further examined. Lower treatment initiation threshold in certain parts of Sweden, due to a combination of different treatment traditions among rheumatologists, and county-specific economic considerations are a possible explanation of geographic area variations in sales of TNF inhibitors in Sweden [35].

Hospital volume and surgeon volume have little effect on 3-year functional outcome following THA [36], but low volume hospitals have an increased revision risk 2, 5, 10 and 15 years follow-up [37]. Additionally high volume hospitals have significantly lower THA related patient injuries [38] and might have lower periprosthetic infection rates [39]. Varagunam and collaborators concluded that are no benefits to patients from centralization of elective surgery into higher volume hospitals regarding PROs [40] and patients can expect similar health improvements, pain reduction, and satisfaction 1 year after a primary THA operation irrespective of years in practice after specialty certification as an orthopaedic surgeon [41]. We did not observe any association between hospital volume and PROs (Additional file 1). Generally the 1-year and 6-year follow-up PRO values show great consistency [42], nevertheless the effect of hospital/surgeon volume on long requires further attention.

Patients in Norrbotten had better results of adjusted EQ-5D, EQ VAS, pain VAS and satisfaction VAS 1 year after THA. Further research is needed to answer if the patients are experiencing a better recovery or if they simply are more satisfied because they had more pain prior to the surgery. Achieving satisfactory pain relief is one of the determining factors of patient satisfaction after lower limb arthroplasty surgery [43], and Norrbotten county ranked high in pain relief. Surgeons’ expectations are predictive of satisfaction 1 year after total hip arthroplasty and information should aim to lower discrepancy between surgeons’ and patients’ expectations [44]. We could not consider this information, as the preoperative education plan is not quantified and registered in Sweden. Patients with low or medium attained education are at risk for less satisfaction with THA [10]. Thus, at least we could conclude that absorption of the information presented by heath care professionals to the patient had an association on the postoperative patient satisfaction. Uniformization of the preoperative education might be a step towards evening out regional differences in postoperative PROs.

In the community of orthopaedic medicine there is an on-going discussion about timing of surgery [45]. This study can contribute to the current knowledge gap by identifying counties that have patients who both have HRQoL and pain within the expected range before surgery and have better results on all postoperative PROs than expected 1 year after the surgery. Further research could then help to identify if the care process and patients’ well-being within these counties is different from other counties. Such study could consider the severity of OA at time of surgery. Further qualitative research could contribute to the understanding of patients’ and orthopaedics’ perceptions of the care process. On a policy level this could then contribute to the standardization of care within the orthopaedic field that might be necessary for a more equal THA care.

Multivariable analysis showed lower HRQoL after surgery for patients living in Stockholm and Västra Götaland. Mölndals’ hospital (located in Västra Götaland), and Karolinska Hospital (located in the county of Stockholm) are both university hospitals, which often get more complex THA patients. This might be a confounding factor in this study since more complex processes can result in poorer PRO values. Another limitation with this study is that we did not examine the differences within one county. One example is Gothenburg in Västra Götaland, the second largest city of Sweden with both socioeconomically weak and strong areas. Furthermore, some areas in Gothenburg have a high incidence of migrants, who might have poorer health literacy due to language and social barriers to access and receive health care information [46]. Krupic and collaborators have observed that PROs after THA are affected by perception of pre- and perioperative information [47]. The results of that study showed that immigrants more often reported poor information before THA, which was related to poorer postoperative PRO values.

PROMs are subjective measures with the aim of ascertain patients’ view with the care. However, with subjectivity there is always a risk that the patients are influences by many factors when reporting. For example doctors’ approach, treatment, pre –and perioperative information, how media show health care in that specific county, or rehab time in physical therapy.

There might be other socio-demographic factors that we did not have the opportunity to control for, for example, disability, sexual orientation, gender identity and/or country of origin, which might be confounders in this analysis. Krupic and collaborators showed in another study that patients born outside of Sweden had more pain 1 year after THA surgery compared to patients born in Sweden [48]. Statistics Sweden’s division of marital status might not capture every patient who has someone at home who can help them with daily activities, which also could affect HRQoL after THA. For example, cohabiting partner is not outlined as a category in Statistics Sweden.

EQ-5D-3 L is a common and well-known measurement for HRQoL, nevertheless, it might not capture every variation in patients’ HRQoL. From 2017, SHAR collects EQ-5D-5 L, a more refined measure that could capture variations better [49]. More research is needed to understand if the geographical variations showed in this study are a time trend or just a coincidence during the 5 years studied.

## Conclusion

The results of this study demonstrate that geographical variations in PROs after THA exist in Sweden today. Patient-related and socioeconomic factors are part of the explanation but even after controlling for these factors, variations across counties still exist. Likely, structural and process differences such as indication for surgery may have an influence on PROs after surgery. Standardization of care at hospital level may decrease geographical variations in postoperative PROs. More research is needed to find the optimal timing of surgery, which could contribute to patients’ well-being after THA.

## Additional files

Additional file 1: Additional results and sensitivity analyses that strengthen the main results presented in the paper. 1. Illustration of the visual analogue scale for patient satisfaction. 2. Distribution of the outcomes and regression residuals and the robustness of the statistical inference. 3. Association between hospital volume and PROs. 4. Sensitivity analysis for under the assumption that the observed variability is due to chance only. 5. County-wise time trends of the expected and observed PROs between 2018 and 2012. (PDF 957 kb)

## Data Availability

The datasets supporting the conclusions of this article are available in the Secure Online Data Access repository (visit Registercentrum webpage and follow Start > In English > Log in to SODA) which can be accessed after appropriate ethical approval.

## Abbreviations

BMI: Body Mass Index
EQ VAS: EuroQol Visual Analogue Scale
EQ-5D: EuroQol 5 Dimensions
HRQoL: Health-related quality of life
OA: Osteoarthritis
Pain VAS: Pain Visual Analogue Scale
PROMs: Patient Reported Outcome Measures
PROs: Patient Reported Outcomes
R2: Coefficient of determination
Satisfaction VAS: Satisfaction Visual Analogue Scale
SEK: Swedish Krona
SHAR: Swedish Hip Arthroplasty Register
THA: Total Hip Arthroplasty
