# Supplementary material for: Geographical variations in patient-reported outcomes after total hip arthroplasty between 2008 - 2012
Source: BMC Health Serv Res. 2019 May 30;19:343. doi: 10.1186/s12913-019-4171-5 (PMC6543668; doi:10.1186/s12913-019-4171-5)
Supplement: Supplementary file 1 — Additional results and sensitivity analyses that strengthen the main results presented in the paper. 1. Illustration of the visual analogue scale for patient satisfaction. 2. Distribution of the outcomes and regression residuals and the robustness of the statistical inference. 3. Association between hospital volume and PROs. 4. Sensitivity analysis for under the assumption that the observed variability is due to chance only. 5. County-wise time trends of the expected and observed PROs between 2018 and 2012. (PDF 957 kb) [file 12913_2019_4171_MOESM1_ESM.pdf]

APPENDIX 1.

Statisfaction VAS.

The Satisfaction visual analogue scale from the postoperative PROM-questionnaire. The patients are asked to : “ Sett a cross at the at the mark that you think corresponds the level of your satisfaction with the outcome of the surgery. ”

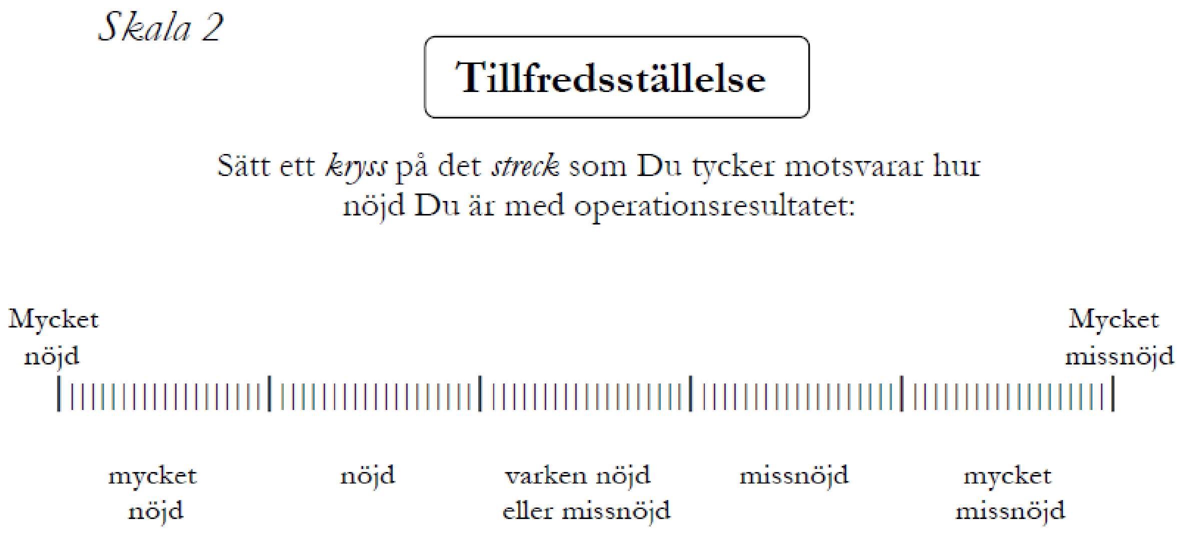

Mycket nöjd	Very satisfied
Nöjd	Satisfied
Varken nöjd eller missnöjd	Neither satisfied or dissatisfied
Missnöjd	Dissatisfied
Mycket missnöjd	Very Dissatified

Assumptions for the multivariable regression analysis.

Ordinary least squares (OLS) or linear least squares is a method for estimating the unknown parameters in a linear regression model. OLS regression assumes that the relationship between the independent and dependent variables are linear, the errors (residuals) and are independent distributed. Specifically, statistical inference the errors should have equal variance and should follow the normal distribution.

In the following we outline the model diagnostics that we conducted to assure the validity of our results. We generally refrained from numerical techniques due to the large sample size, and employed graphical methods which are more flexible.

Distribution of the outcomes, residuals and regression coefficients

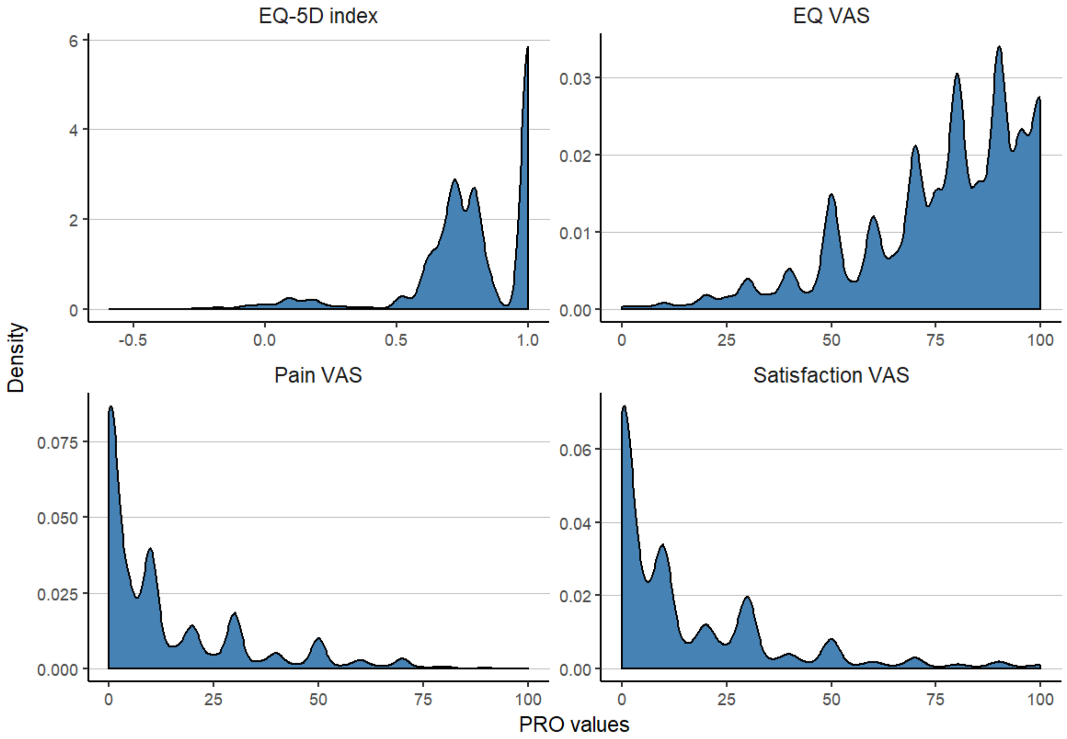

Figure 1. Distribution of the postoperative PRO values

PROMs data is known to violate the normality assumption, and this is illustrated in Figure 1. Perhaps with the exception this violation is translated to the residuals as well, perhaps with the exception of EQ VAS (Figure 2).

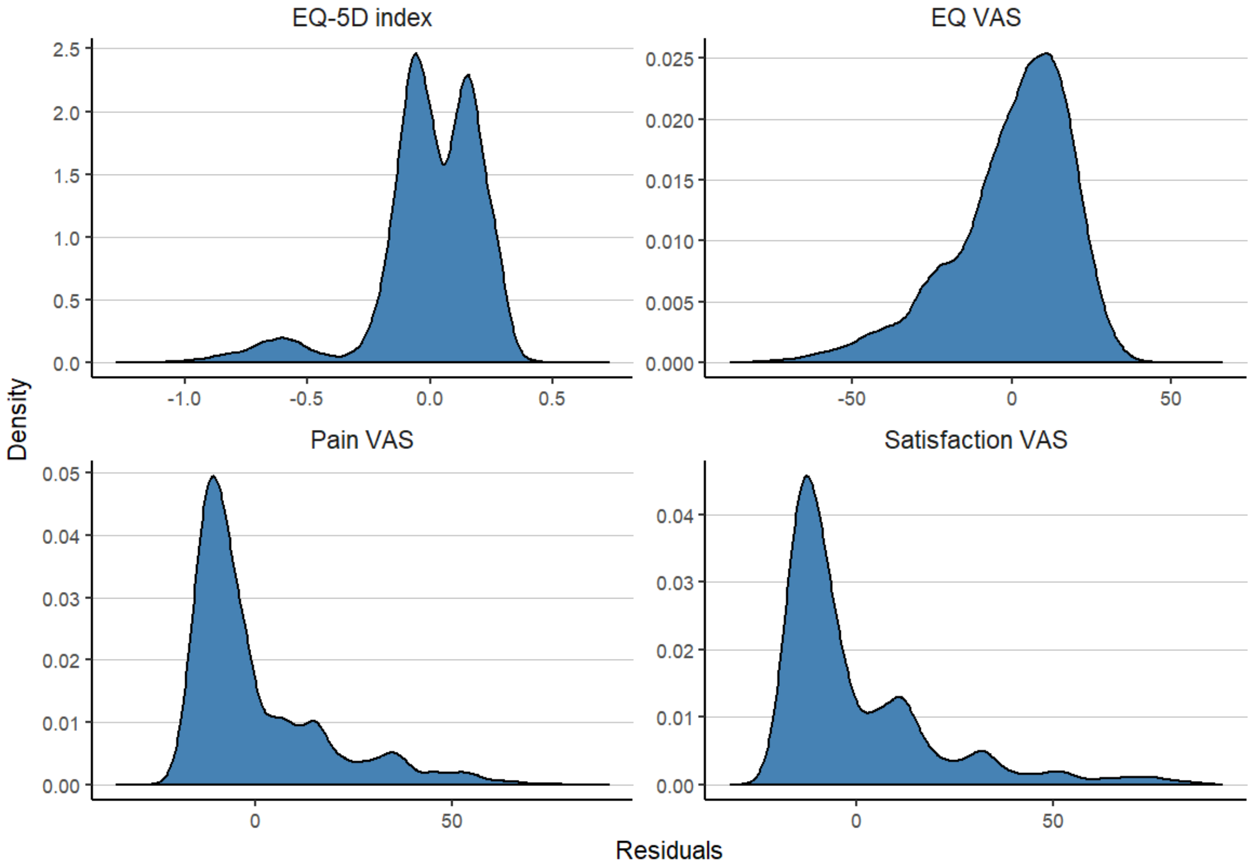

Figure 2. Distribution of the residuals

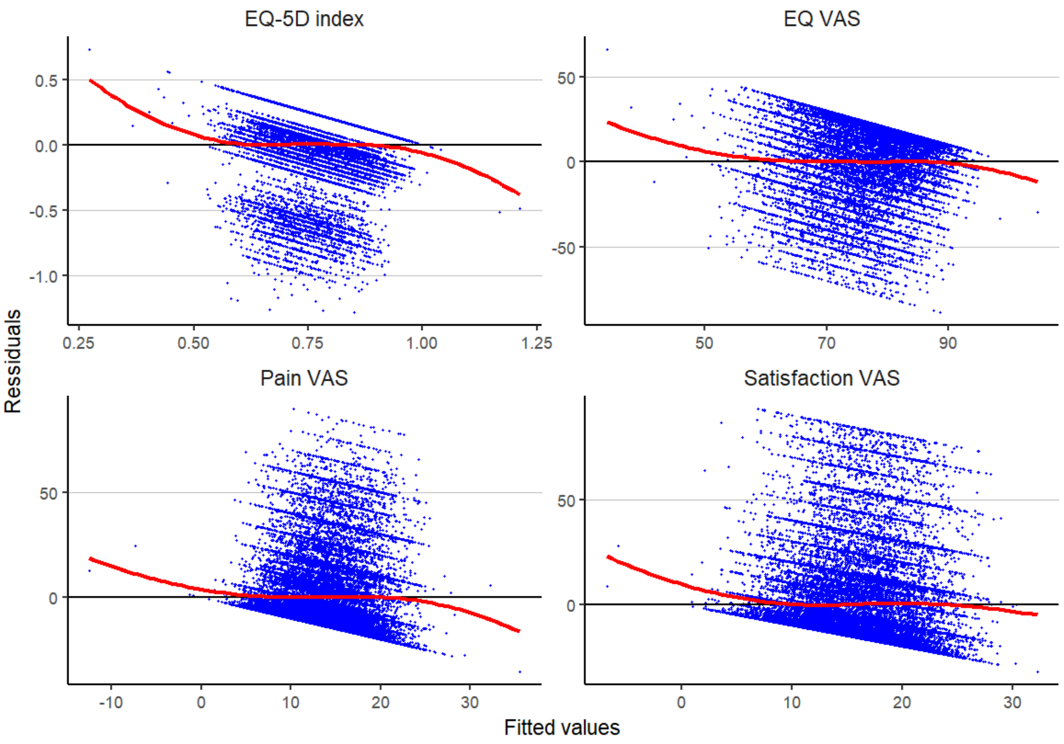

Figure 3. Residuals vs fitted values

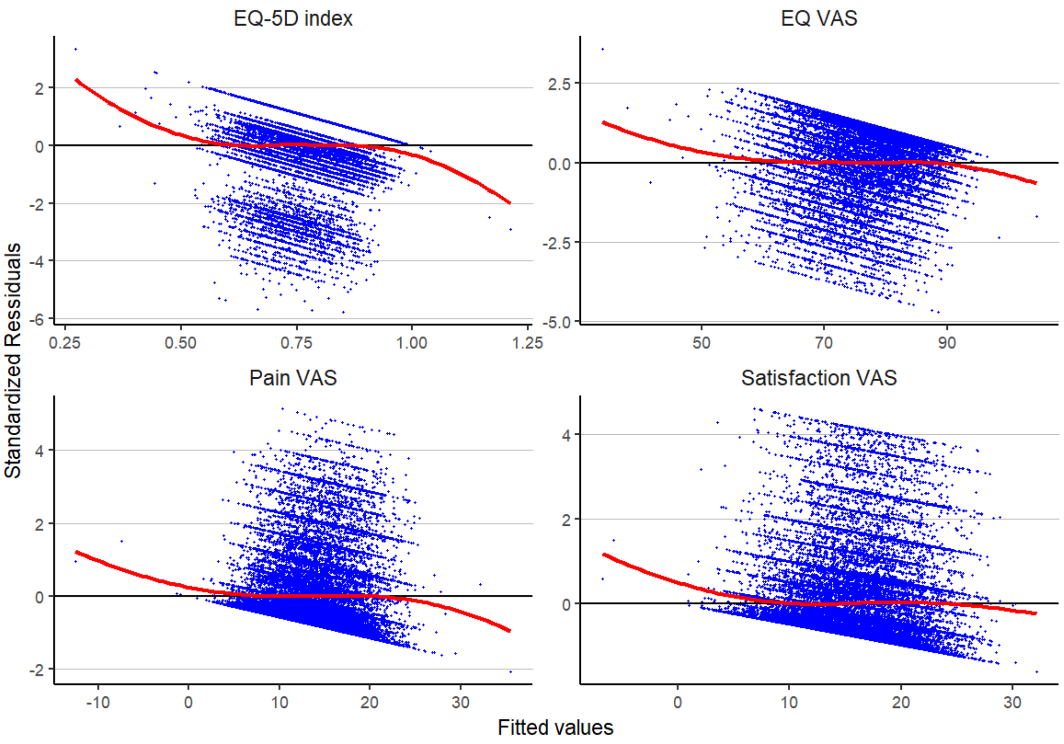

Figure 4. Studentized residuals vs fitted values

With the exception of a few outliers the residuals are scattered around zero and there are no apparent indications for non-constant variance or nonlinearity (Figure 3 & 4). True, the

dominance of categorical independent variables results in clustering of the plotted values, making interpretation difficult.

To assure that estimation and statistical inference are valid we used resampling methods. We used non-parametric bootstrapping with 1000 resamples to estimate the empirical distribution of the regression coefficients and compared the confidence intervals based on the normality assumption with the bootstrap confidence intervals.

The sheer number of coefficients does not allow presentation of the results for all coefficients, thus we randomly chose (using the r code `sample(1:20, 1)` to pick a county) Uppsala county. Using the estimated regression coefficient and associated standard we plotted a normal density line error we compared the empirical distribution of the coefficients.

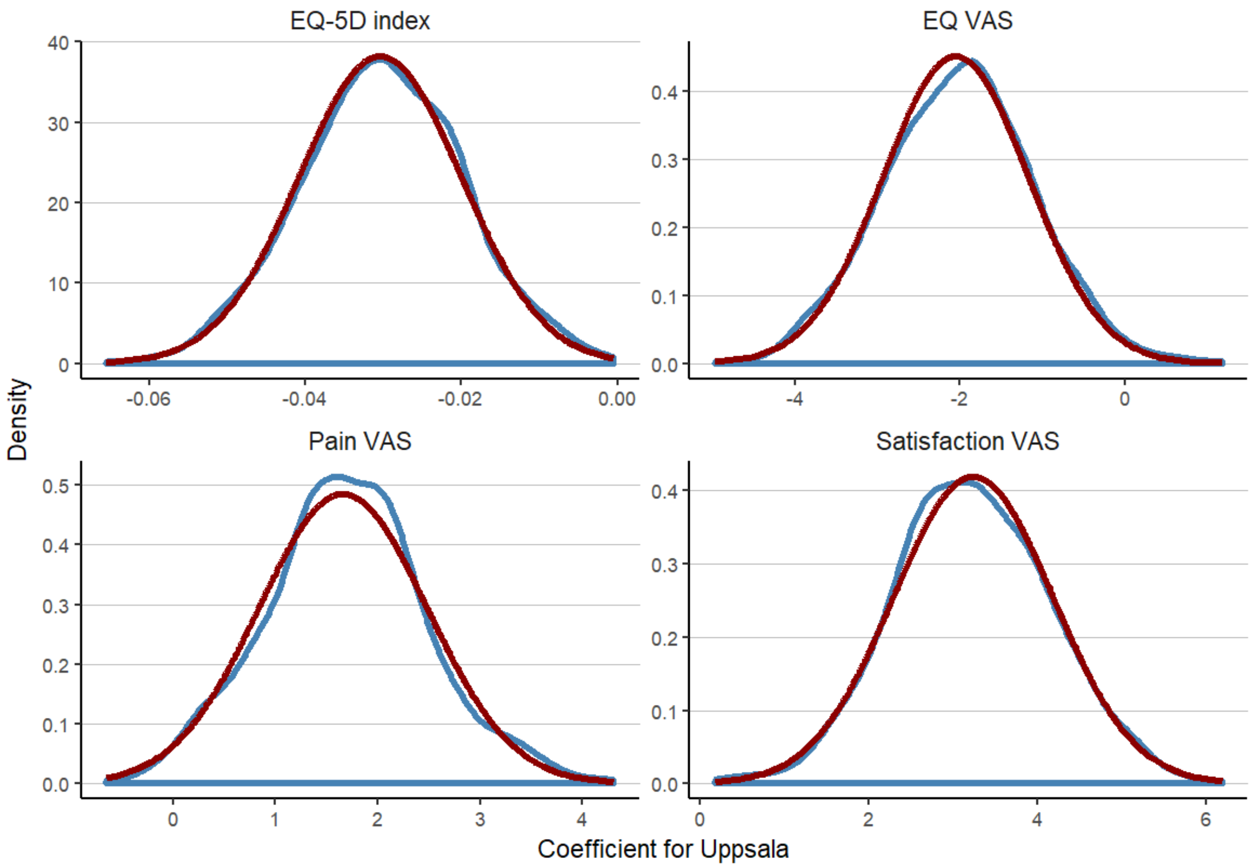

Figure 5. Empirical (blue line) and theoretical distribution (red line) of the regression coefficients for Uppsala County.

The theoretical and empirical distribution largely overlapped. Additionally statistical inference based on asymptotic normality and resampling were in complete agreement (Figure 2).

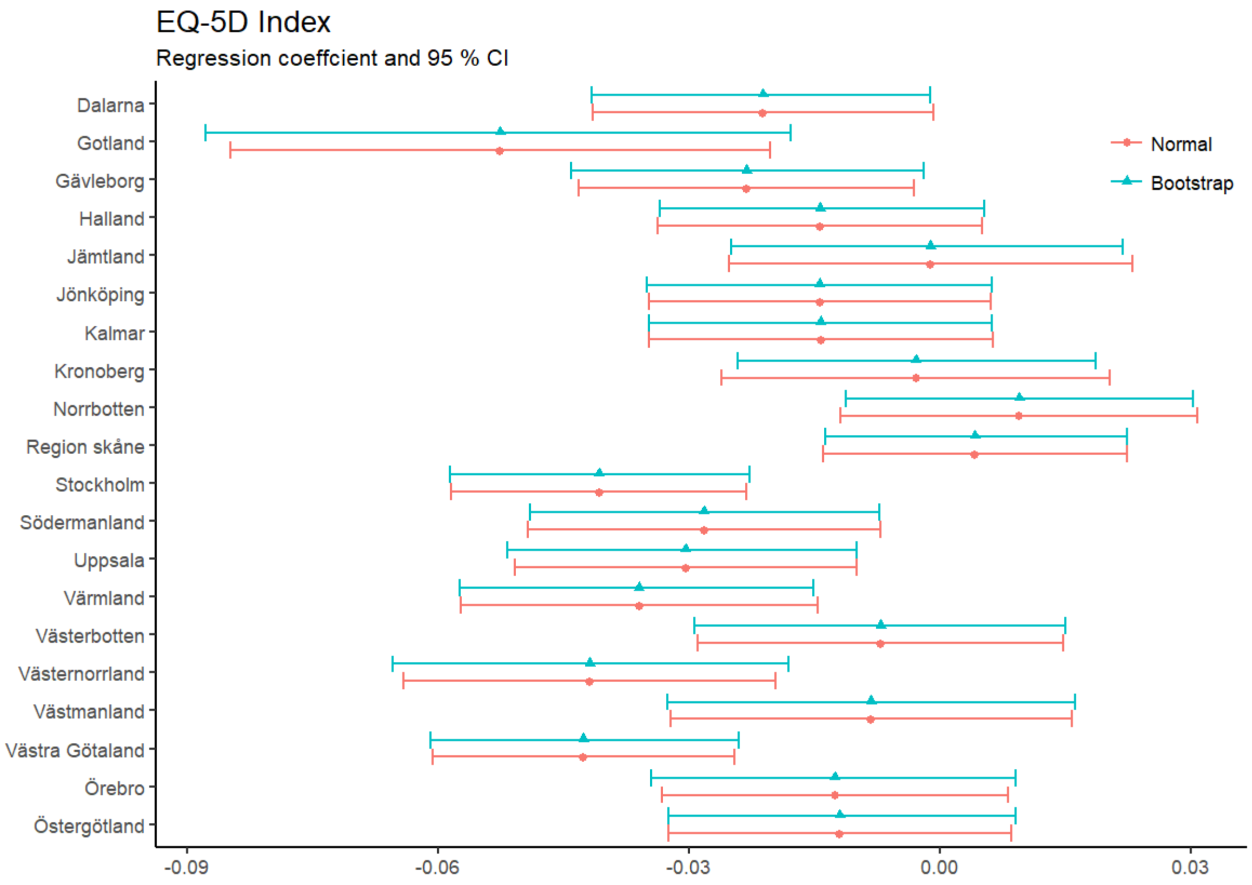

Figure 6. Regression coefficients for the association between county and postoperative EQ-5D index based on the assumption of asymptotic normality and non-parametric bootstrapping.

Hospital volume vs PROs

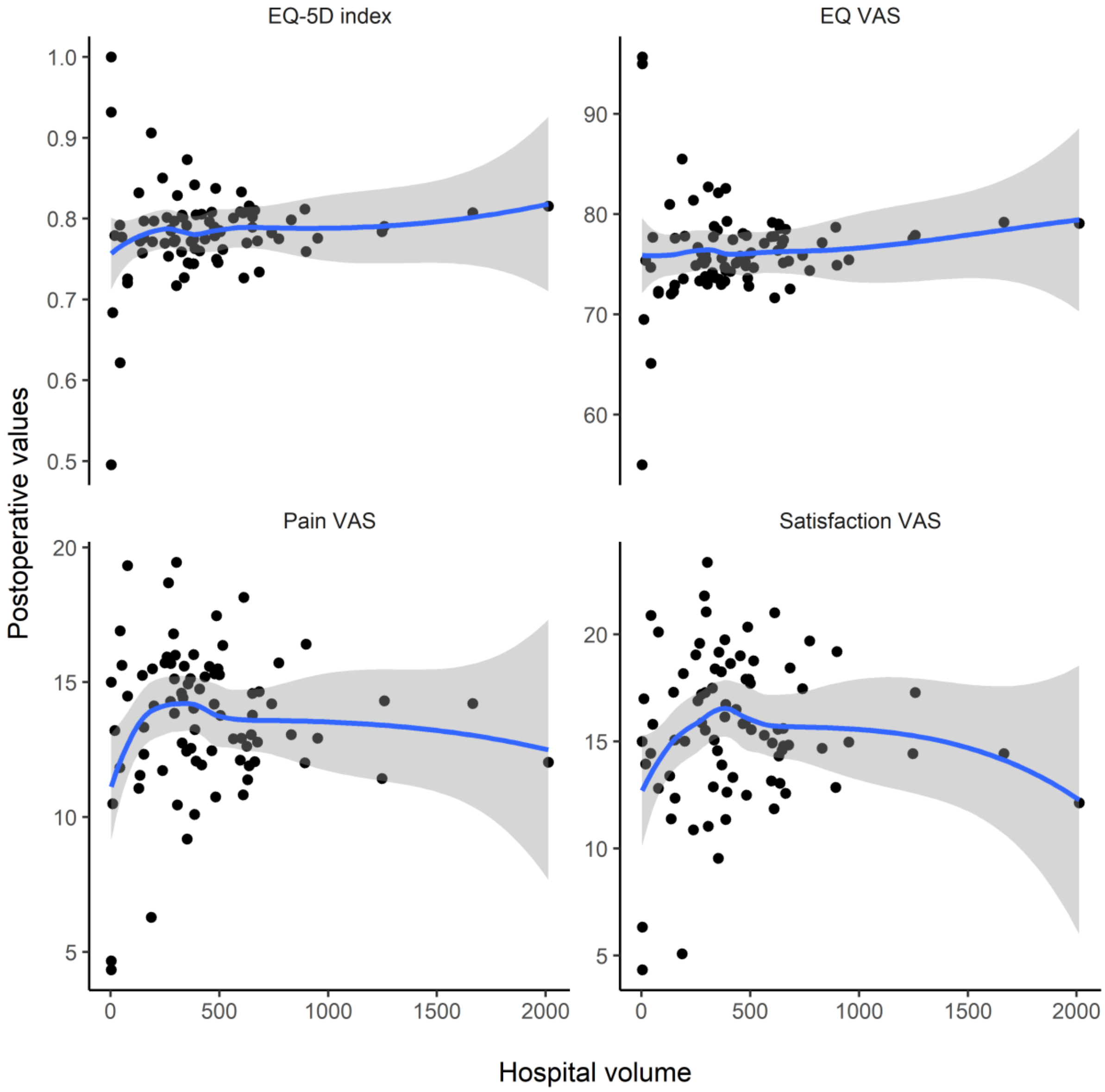

Figure 7. Association between hospital volume and postoperative PROM values.

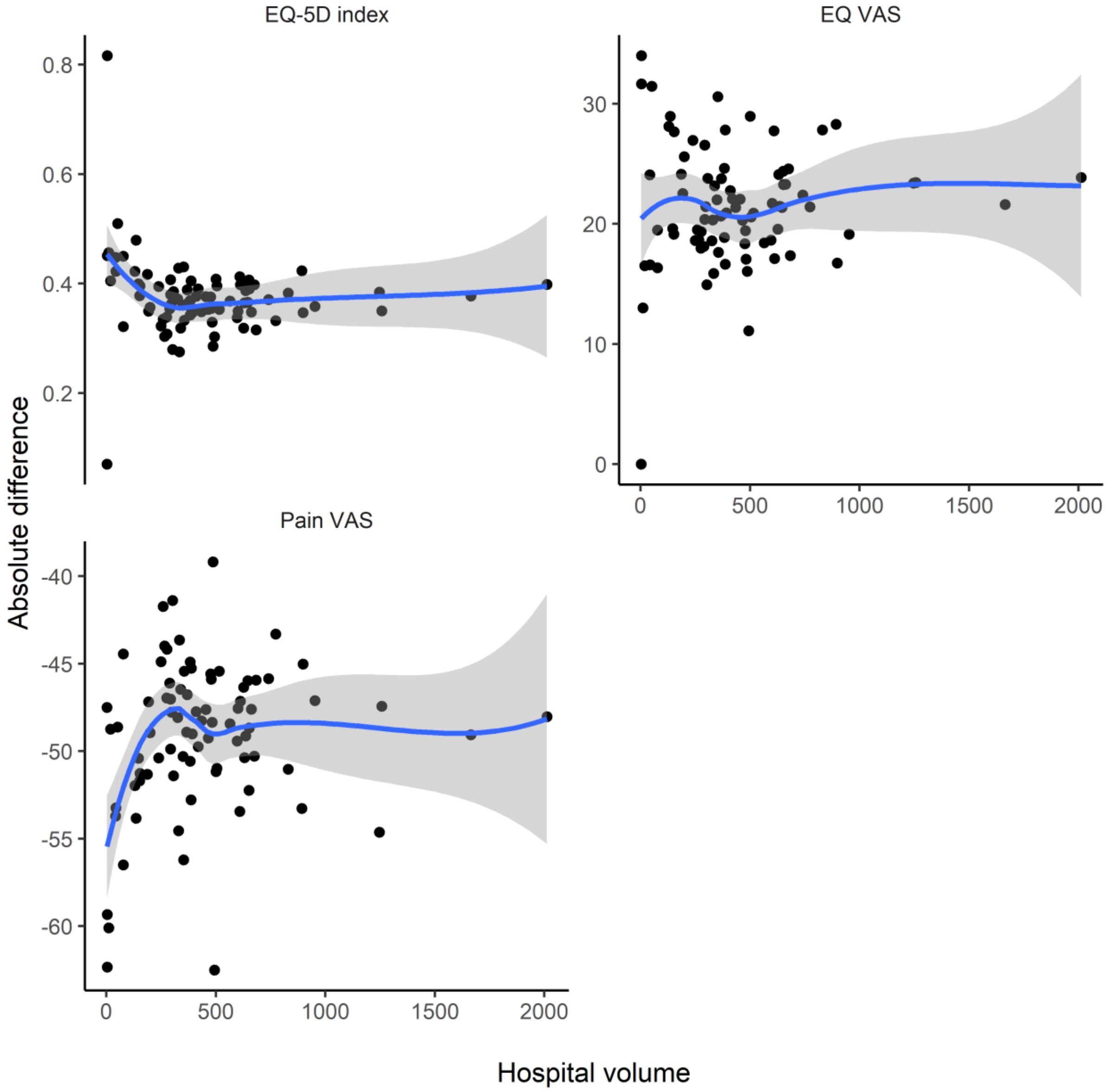

Figure 8. Association between hospital volume and absolute differences in postoperative and preoperative PROM values.

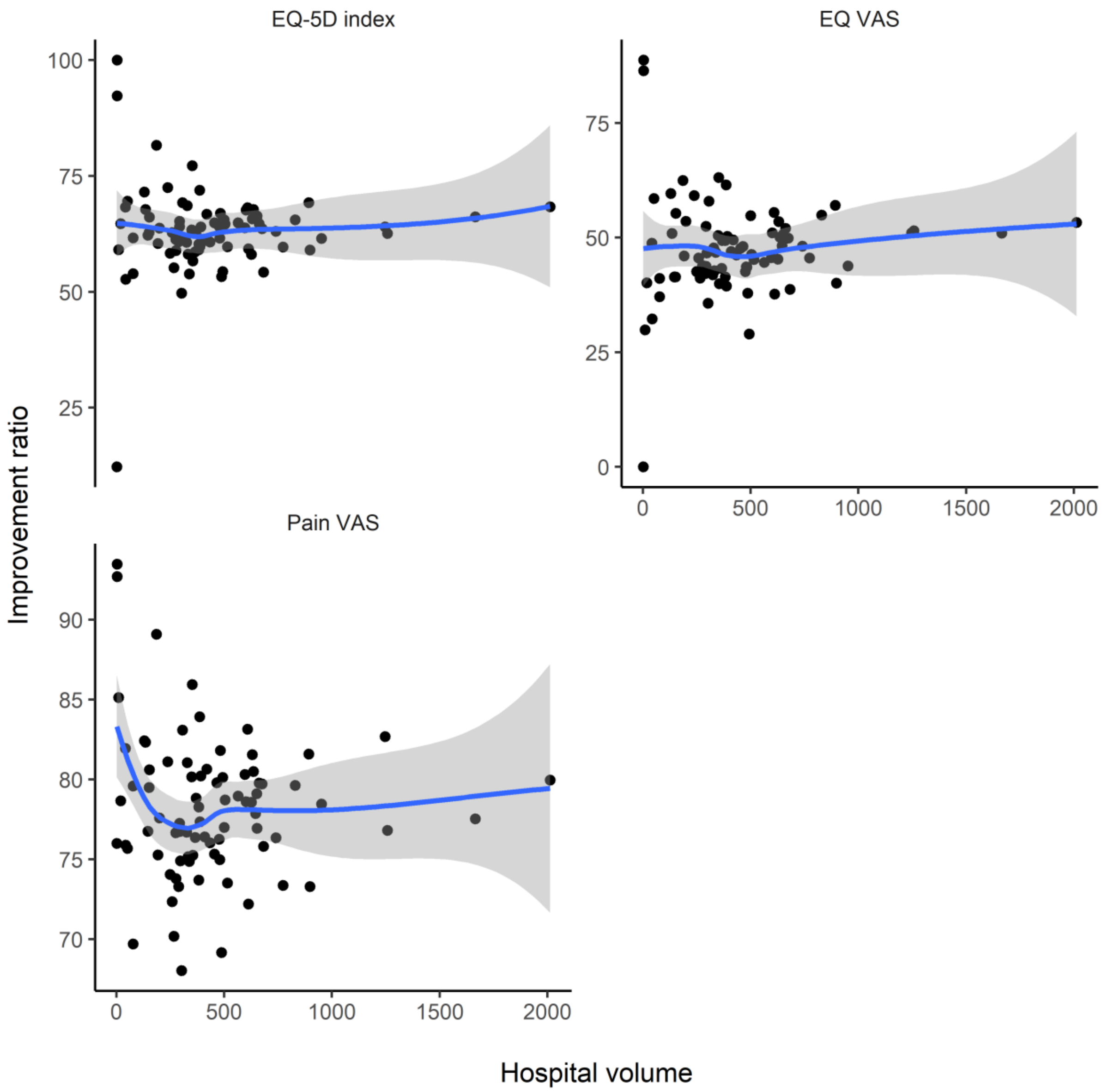

Figure 9.

Is the observed variability due to random sampling?

One important question that is need to be addressed is if the observed county-wise variability is due to sampling variability or there is true systematic deviations.
In a hypothetical world where all counties and all patients would be exactly the same we would still observe variation due to chance and sampling variability.
Naturally counties and patients differ but it is important to see if the observed variability can be attributed sampling variability.
In order to examine this issue we undertake used permutations. Patients were randomly assigned to counties, the assignment took into consideration the number of patients operated in each county.
After this random assignment was done the analysis were repeated and difference between the expected and observed PRO values where calculated. This procedure was repeated 1000 times.
The results were unanimous and for each PRO measure considered the observed county-wise variability were substantially larger than one would expect do to random sampling (Figure 7).
Both the expected and observed values are centred on 0, however the standard deviation for the observed values (0.0168) vas approximately by factor 67 larger than for the expected values

(0.0002) for the EQ-5D index. For EQ VAS was the observed values (1.468) approximately by factor 95 larger than for the expected values (0.015). Corresponding observed values for Pain VAS was 1.141 which was approximately by factor 60 larger than for the expected values (0.019). The standard deviation for the observed values (1.857) was approximately by factor 135 larger than for the expected values (0.013) for Satisfaction VAS.

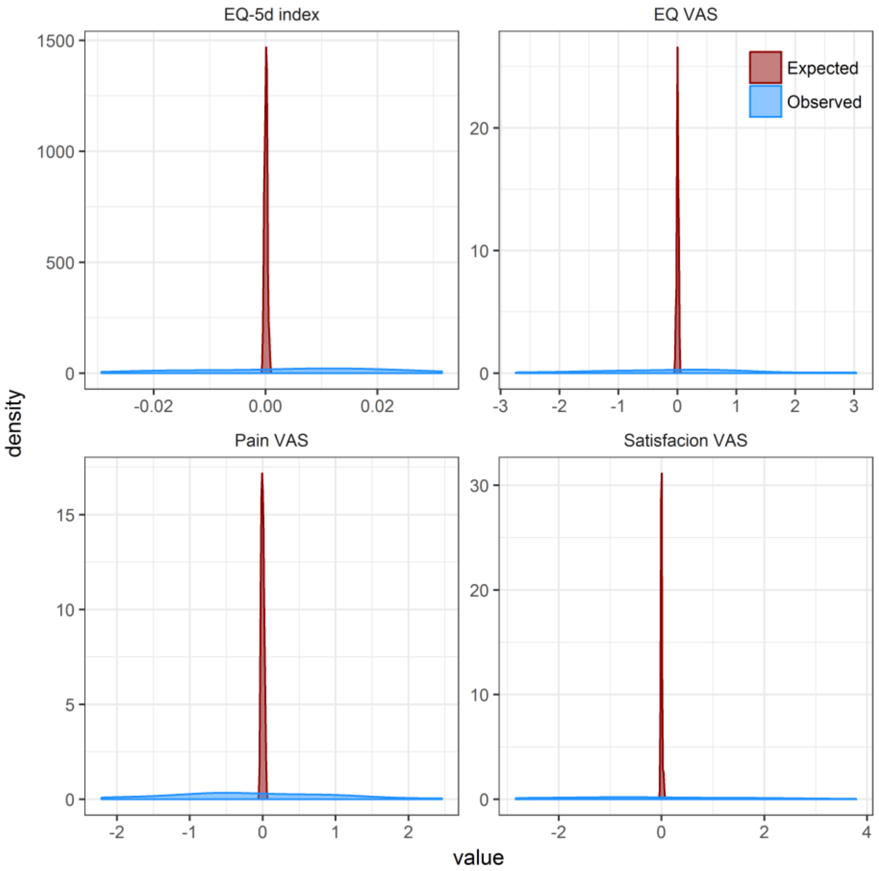

Figure 7. Expected and observed county-wise variability of the PRO measures.

Time trends in PROs

Figure 6. Trends in postoperative PROs between 2008 and 2012. The figures present the evolution of the PRO values for the 21 Swedish counties. The expected value was calculated using a multivariable regression equation.
Some counties like Gotland or Västmanland (among others) exhibited higher year to year variation and inconsistent trends, while others like Västra Gotaland, Örebro or Uppsala showed clearer trends and consistent year to year results. Generally counties with larger population and higher operation volumes had more consistent trends, while counties with lower population and operation volumes varied more considerably.

Blekinge

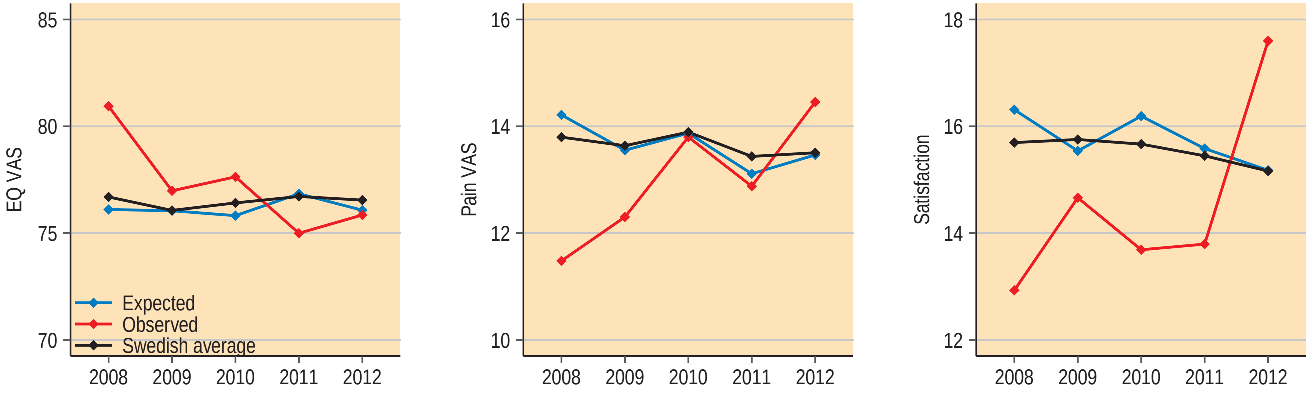

Dalarna

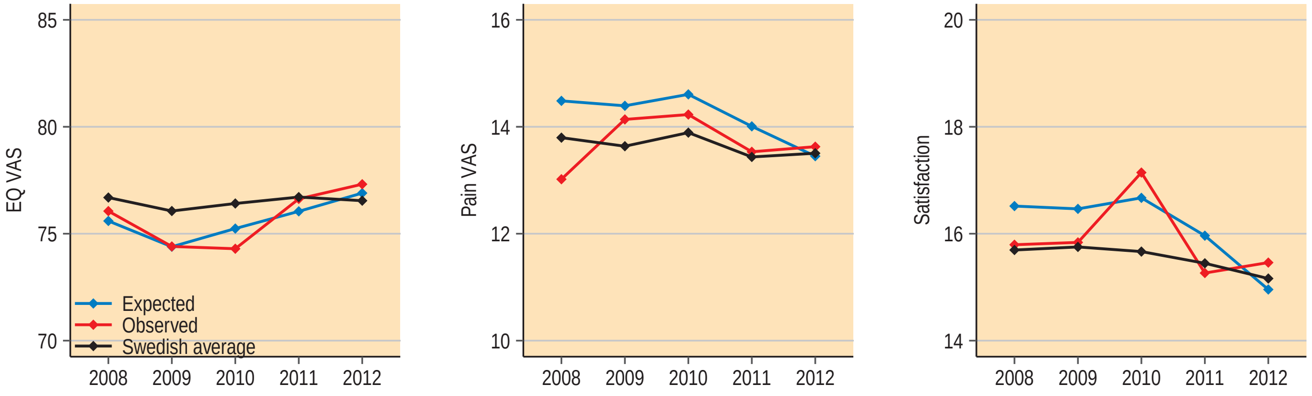

Gotland

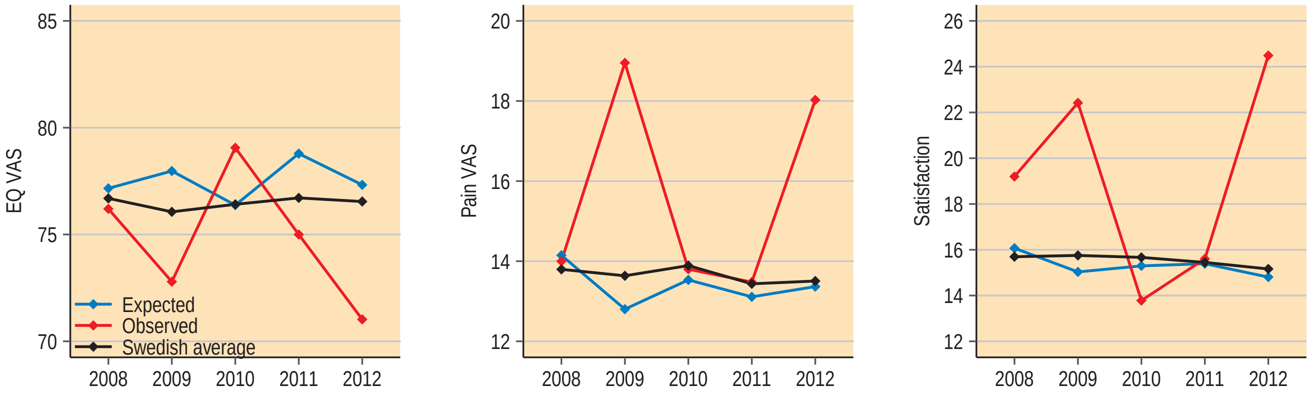

Gävleborg

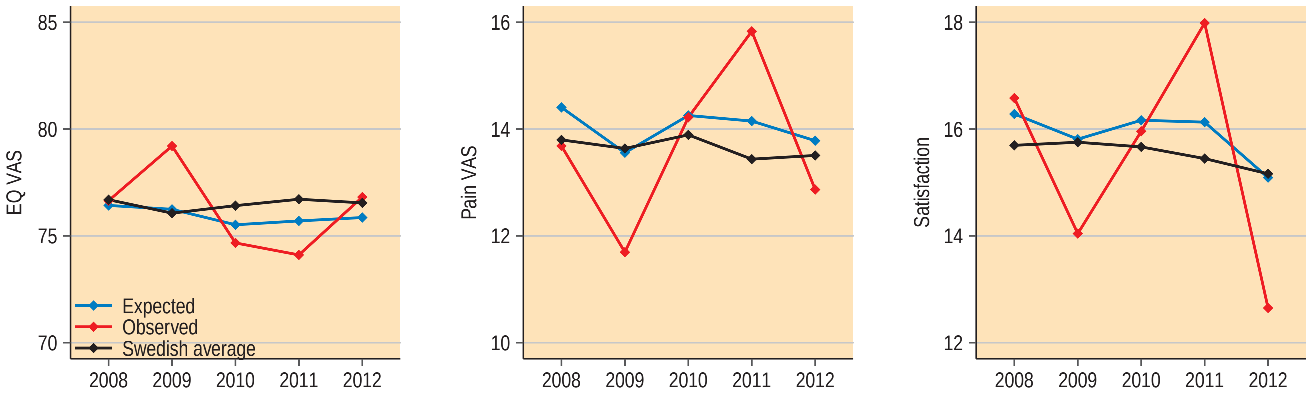

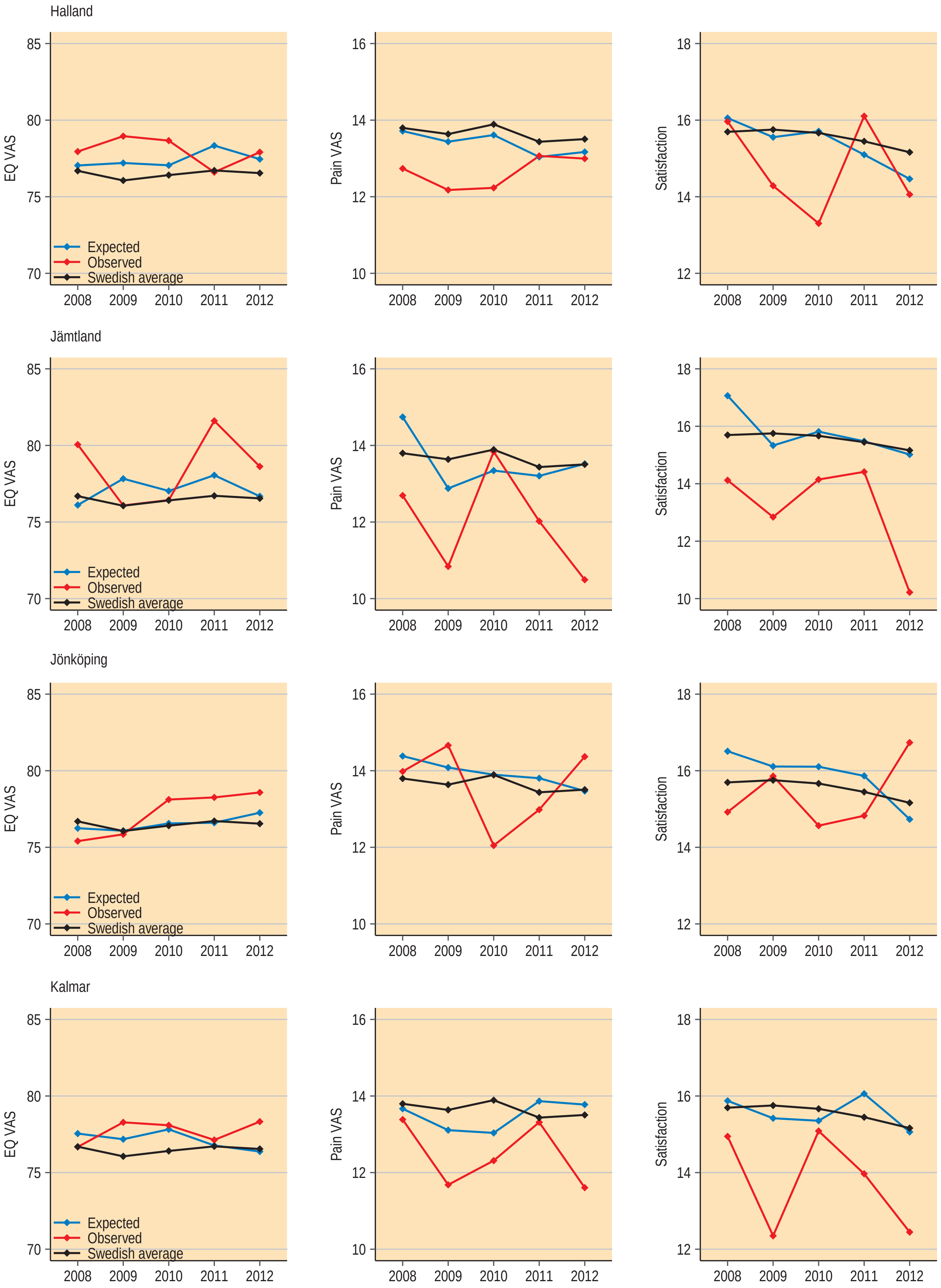
Halland
Jämtland
Jönköping
Kalmar
EQ VAS
Pain VAS
Satisfaction
Expected
Observed
Swedish average
70
75
80
85
10
12
14
16
18
2008
2009
2010
2011
2012

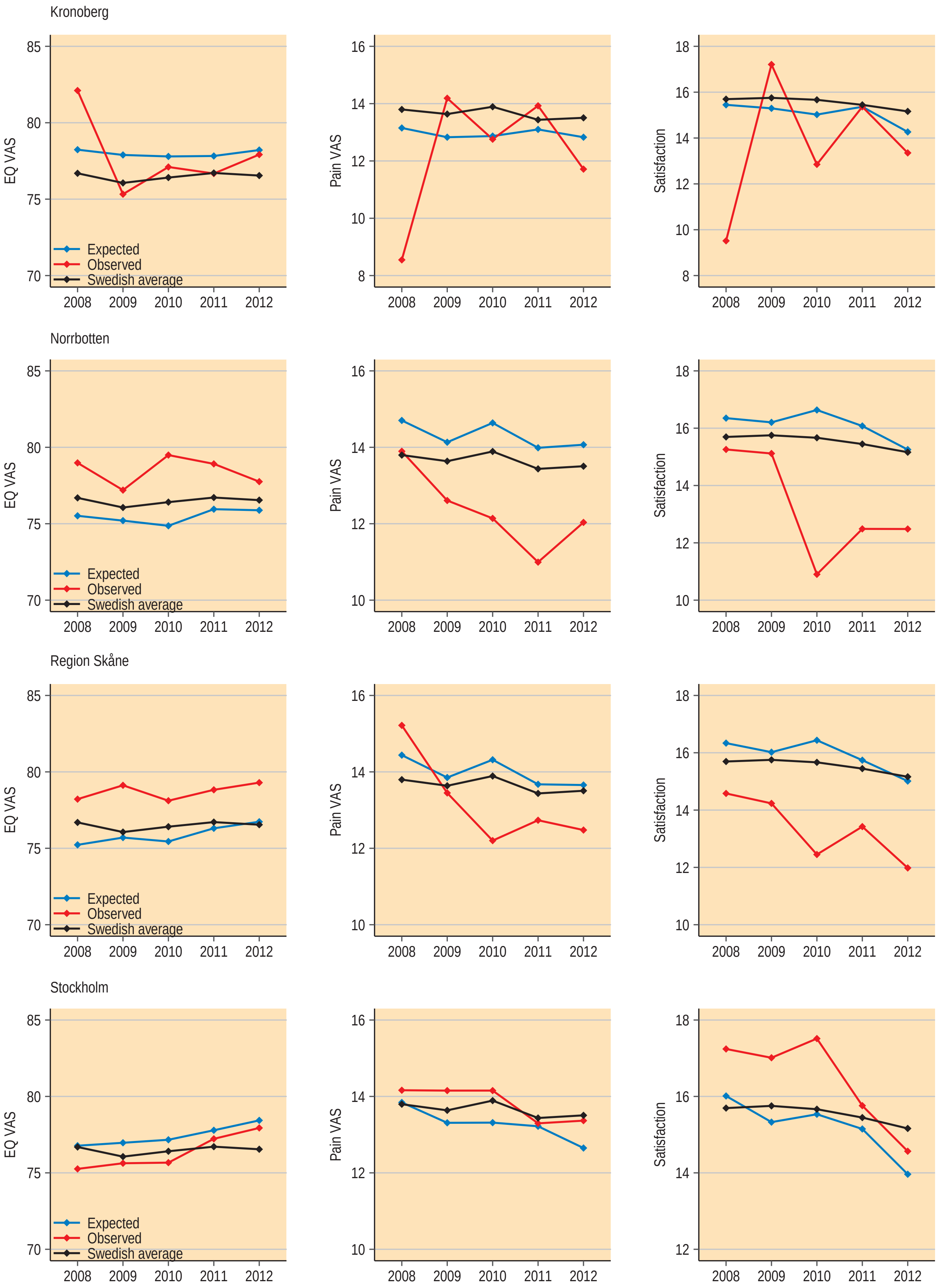
Kronoberg
Norrbotten
Region Skåne
Stockholm
EQ VAS
Pain VAS
Satisfaction
Expected
Observed
Swedish average
2008
2009
2010
2011
2012

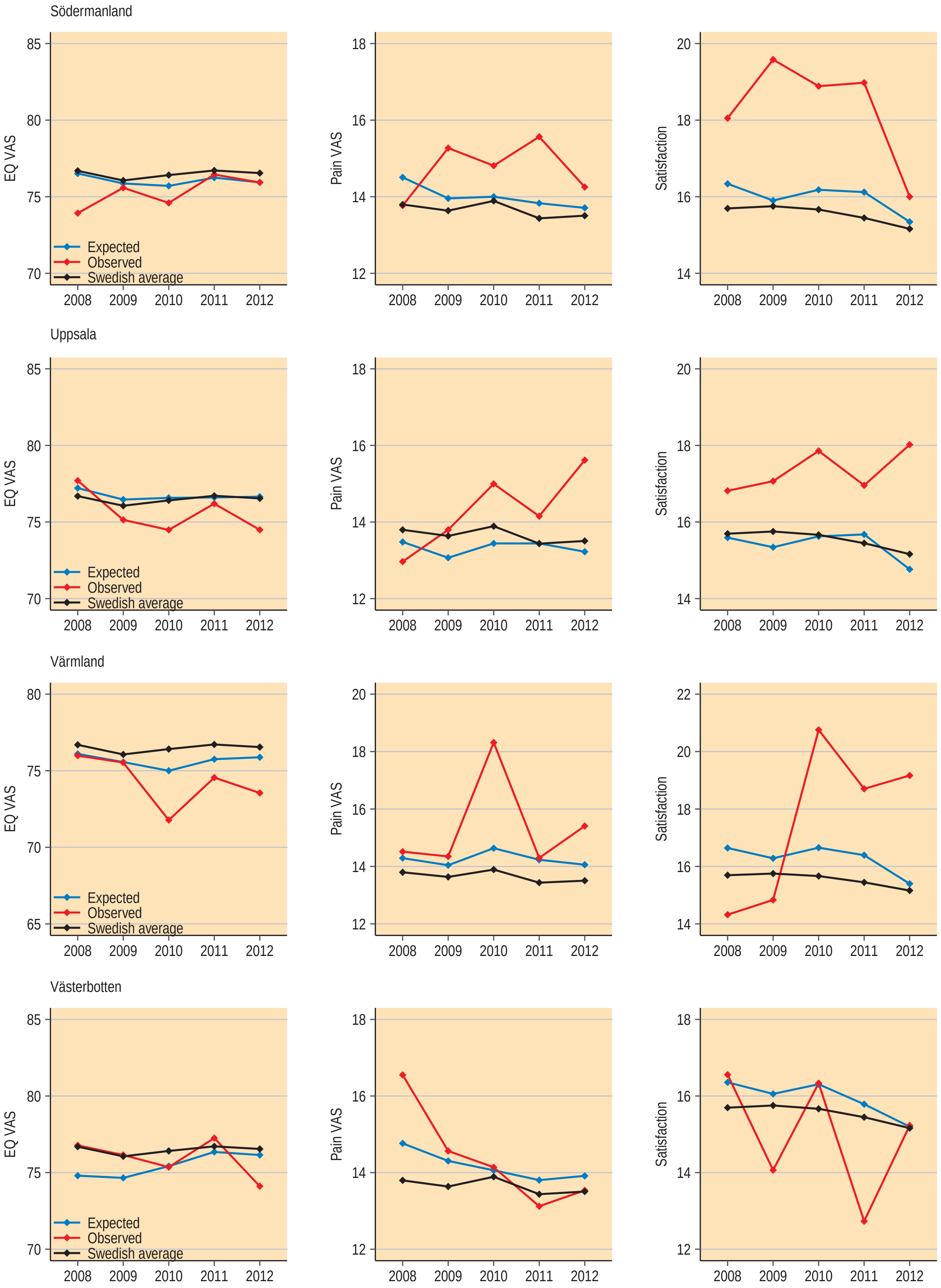
Södermanland
Uppsala
Värmland
Västerbotten
EQ VAS
Pain VAS
Satisfaction
Expected
Observed
Swedish average
2008
2009
2010
2011
2012

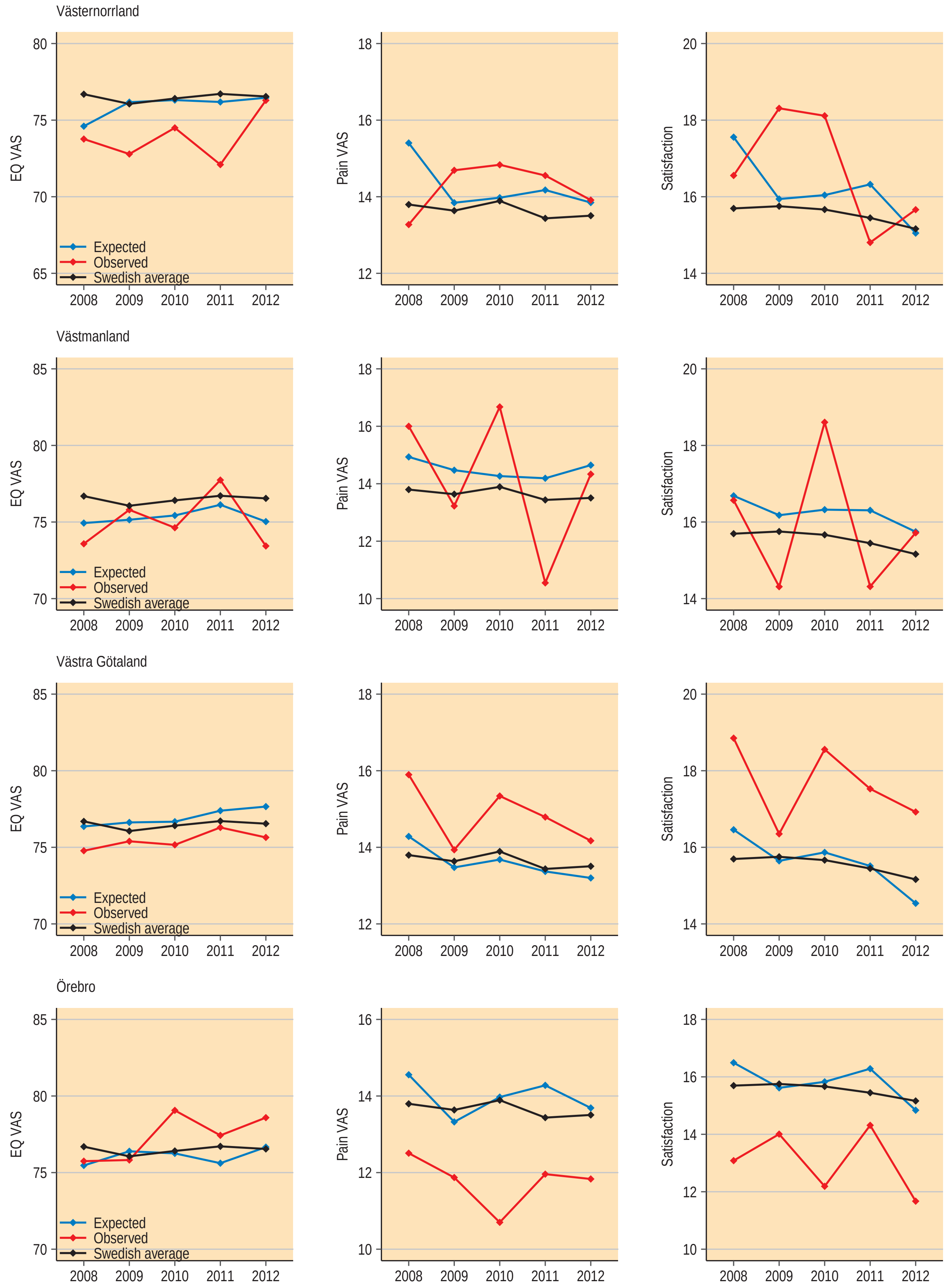
Västernorrland
Västmanland
Västra Götaland
Örebro
EQ VAS
Pain VAS
Satisfaction
Expected
Observed
Swedish average
2008
2009
2010
2011
2012

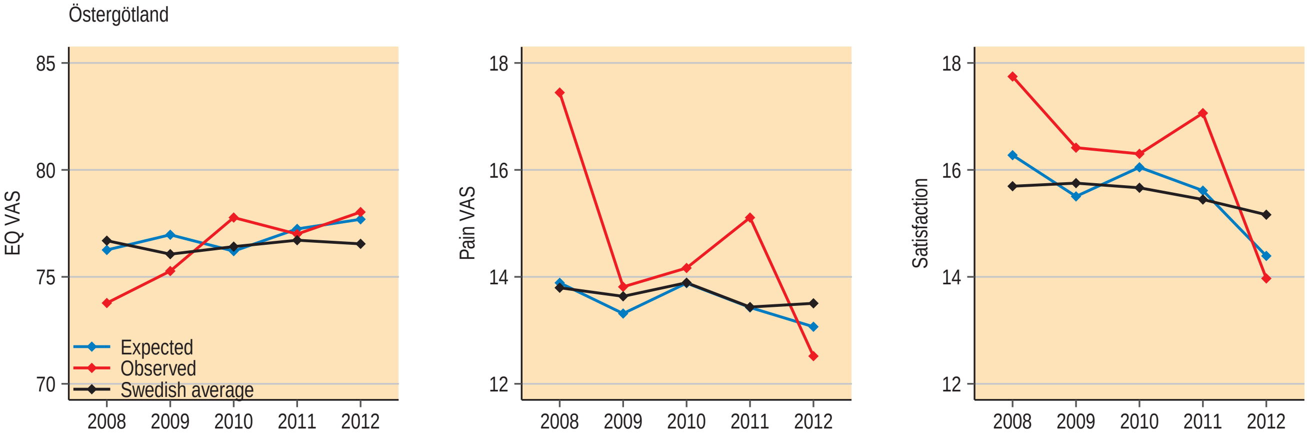
Östergötland
85
80
75
70
EQ VAS
Expected
Observed
Swedish average
2008
2009
2010
2011
2012
18
16
14
12
Pain VAS
18
16
14
12
Satisfaction
